# Discovery of PRDM16‐Mediated TRPA1 Induction as the Mechanism for Low Tubulo‐Interstitial Fibrosis in Diabetic Kidney Disease

**DOI:** 10.1002/advs.202306704

**Published:** 2023-12-10

**Authors:** Fang Xu, Hongwei Jiang, Xiaozhou Li, Jian Pan, Huiling Li, Luxiang Wang, Pan Zhang, Junxiang Chen, Shuangfa Qiu, Yuxin Xie, Yijian Li, Dongshan Zhang, Zheng Dong

**Affiliations:** ^1^ Department of Emergency Medicine Second Xiangya Hospital Central South University Changsha Hunan 410011 P. R. China; ^2^ Emergency Medicine and Difficult Diseases Institute Second Xiangya Hospital Central South University Changsha Hunan 410011 P. R. China; ^3^ Department of Nephrology Second Xiangya Hospital Central South University Changsha Hunan 410011 P. R. China; ^4^ Department of Endocrinology First Affiliated Hospital of Henan University of Science and Technology Luoyang Henan 471000 P. R. China; ^5^ Department of Ophthalmology Central South University Changsha Hunan 410011 P. R. China; ^6^ Department of Epidemiology and Health Statistics Xiangya School of Public Health Central South University Changsha Hunan 410011 P. R. China; ^7^ Department of Urology Second Xiangya Hospital Central South University Changsha Hunan 410011 P. R. China; ^8^ Department of Cellular Biology and Anatomy Medical College of Georgia at Augusta University Augusta Georgia 30906 USA

**Keywords:** diabetic kidney disease, MAPK, PRDM16, TGF‐β1, TRPA1, tubulo‐interstitial fibrosis

## Abstract

The pathogenesis of Diabetic kidney disease(DKD) involves pathological changes in both tubulo‐interstitium and the glomerulus. Surprisingly, tubulo‐interstitial fibrosis (TIF), does not develop significantly until the late stage of DKD. Here, it is demonstrated that PR domain‐containing 16 (PRDM16) is a key to the low level of TIF in DKD. In the experiments, PRDM16 is upregulated in high glucose‐treated renal tubular cells, DKD mouse kidneys, and renal biopsy of human DKD patients via activation of NF‐κB signal pathway. High glucose‐induced expression of fibrotic proteins in renal tubular cells is suppressed by PRDM16. Mechanistically, PRDM16 bound to the promotor region of Transient receptor potential ankyrin 1 (TRPA1) to transactivate its expression and then suppressed MAPK (P38, ERK1/2) activation and downstream expression of TGF‐β1. Knockout of PRDM16 from kidney proximal tubules in mice blocked TRPA1 expression and enhanced MAPK activation, TGF‐β1 production, TIF development, and DKD progression, whereas knock‐in of PRDM16 has opposite effects. In addition, overexpression of PRDM16 or its induction by formononetin ameliorated renal dysfunction and fibrosis in db/db diabetic mice. Finally, the above finding are detected in renal biopsies of DKD patients. Together, these results unveil PRDM16/TRPA1 as the mechanism responsible for the low level of TIF in the early stage of DKD by suppressing and TGF‐β1 expression.

## Graphical abstract

aPRDM16 is induced in DKD models and human DKD patients. Upon induction, PRDM16 suppresses renal fibrosis. Mechanistically, PRDM16 transactivates TRPA1, which suppresses the pro‐fibrotic pathway of MAPK /TGF‐β1. Thus, the work has unveiled an anti‐fibrosis mechanism that is activated in renal tubular cells to keep TIF during the development of DKD, suggesting new therapeutic targets and strategies.

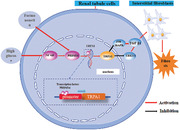

## 1 Introduction

One in 10 adults worldwide now live with diabetes that is associated with serious complications, including diabetic kidney disease (DKD). One‐third of diabetes patients develop DKD, which accounts for almost one‐half of cases of chronic kidney disease (CKD) and end‐stage renal disease (ESRD).^[^ ^1^, ^2^, ^3^, ^4^ ^]^ The pathogenesis of DKD involves not only glomerular dysfunction but also tubulointerstitial pathologies.^[^ ^2^, ^3^, ^4^ ^]^ However, the molecular mechanism of tubulointerstitial pathologies in DKD remains largely unclear.

Tubulo‐interstitial fibrosis (TIF) is a pathological feature of CKD that is characterized by the deposition of extracellular matrix in the interstitial space. TIF is also considered to be the final common pathway to the complete loss of renal function resulting in ESRD.^[^ ^5^, ^6^ ^]^ In this regard, DKD is surprisingly unique, because TIF is not obvious during the initiation or progression of DKD and only becomes notable at the late stage of the disease.^[^ ^2^, ^3^, ^4^, ^7^ ^]^ In contrast, DKD is associated with other tubulo‐interstitial pathologies, including tubular hypertrophy followed by degeneration. The reason for low TIF in the early stage of DKD is completely unknown.

PRD1‐BF1‐RIZ1 homologous domain‐containing protein 16 (PRDM16) is a zinc‐finger transcription factor originally cloned from a chromosomal translocation in patients with acute myeloid leukemia and myelodysplastic syndrome.^[^ ^8^ ^]^ PRDM16 is a strong driver of brown/beige fat biogenesis.^[^ ^9^, ^10^, ^11^, ^12^, ^13^ ^]^ Interestingly, PRDM16 has been implicated in adipose tissue fibrosis.^[^ ^14^, ^15^ ^]^ The role and regulatory mechanism of PRDM16 in renal pathophysiology including DKD and renal fibrosis is currently unknown.

In the present study, we show that PRDM16 is induced in DKD models and in renal biopsies of human DKD patients. Upon induction, PRDM16 suppresses renal fibrosis. Mechanistically, PRDM16 transactivates TRPA1, which then suppresses the pro‐fibrotic pathway of MAPK (P38 and ERK1/2)/TGF‐β1. Thus, the work has unveiled an intrinsic anti‐fibrosis mechanism that is activated in renal tubular cells to keep TIF at a low level during the development of DKD, suggesting new therapeutic targets and strategies.

## 2 Results

### 2.1 PRDM16 is Induced in DKD Models and in Human DKD Renal Biopsies, and the Involvement of NF‐ κ B

**Figure 1:**
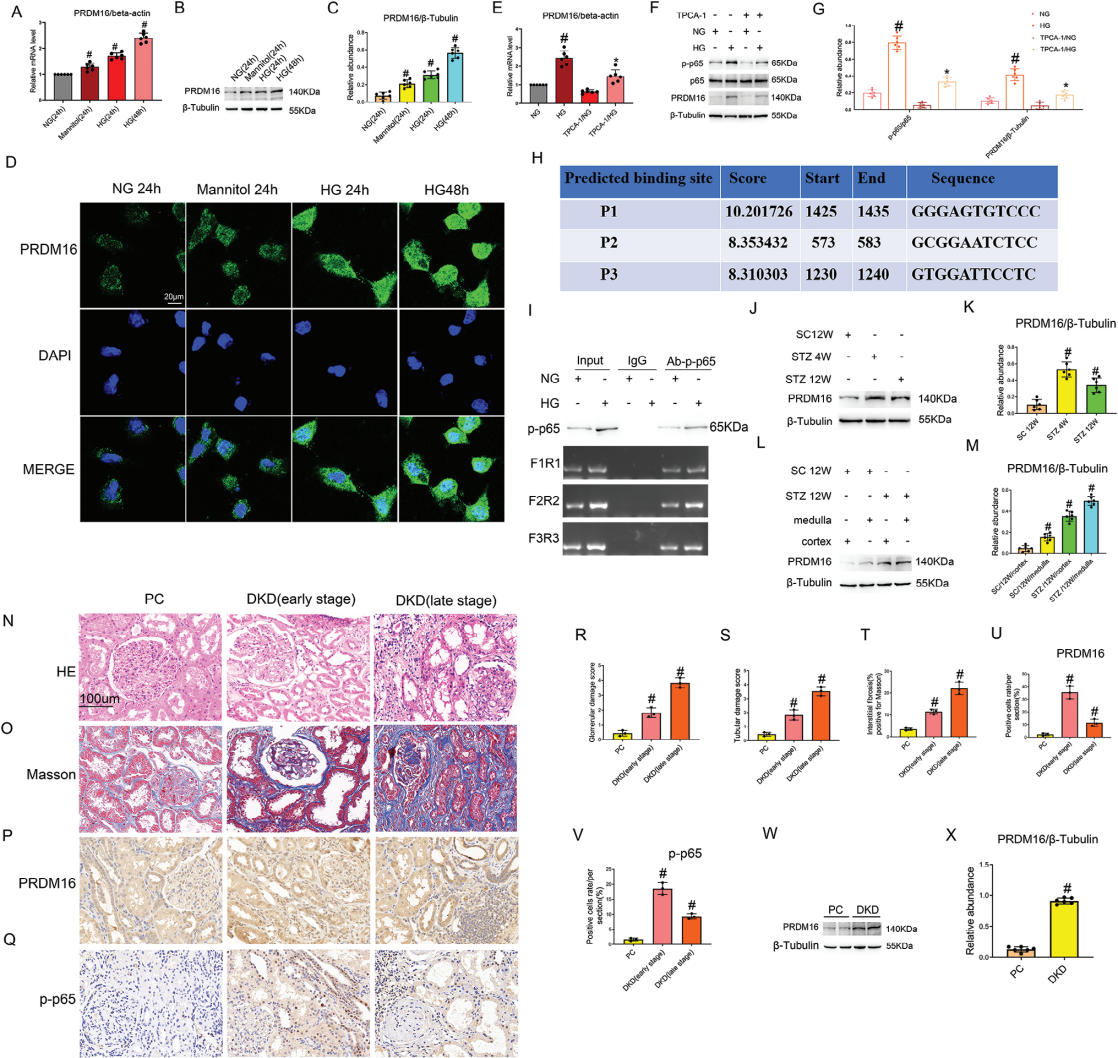
PRDM16 is induced in DKD models and in human DKD renal biopsies, and the involvement of NF‐κB. A–D) BUMPT cells were incubated with NG (5 mmol L^−1^ D‐glucose), mannitol (5 mmol L^−1^ D‐glucose+25 mmol L^−1^ D‐mannitol) or HG (30 mmol L^−1^ D‐glucose) for 24–48 h. (A) RT‐qPCR analysis of the PRDM16 mRNA expression. (B) Immunoblot analysis of PRDM16 protein expression. (C) Densitometry analysis of immunoblot bands. (D) Immunofluorescence detection of the expression and localization of PRDM16 in BUMPT cells. E–G) BUMPT cells were treated with 100um TPCA1 (NF‐κB inhibitor), and then subjected to HG or NG incubation. (E) RT‐qPCR analysis of the PRDM16 mRNA expression. (F) Immunoblot analysis of PRDM16 and p‐p65 protein expression. (G) Densitometry analysis of immunoblot bands. H,I) BUMPT cells were subjected to NG or HG treatment for 48 h for ChIP assay using p‐p65 antibody. (H) NF‐κ B binding site on PRDM16 gene promoter predicted by JASPAR database. (I) ChIP assays showing the binding of NF‐ κ B to the P1, P2, and P3 sites of PRDM16 gene promoter. J–M) C57BL/6J mice were intraperitoneally injected with 50 mg kg^−1^ body weight STZ for 5 consecutive days or injected with sodium citrate (SC) as a control. The fasting blood glucose levels of more than 200 mg dL^−1^ for two consecutive measurements were regarded as diabetic. (J, L) Immunoblot analysis showing PRDM16 induction in diabetic kidney tissues. K,M) Densitometry analysis of immunoblot bands. N–V) Human kidney samples from DKD (early stage) and DKD (late stage) patients and paracancerous (PC) normal kidney tissues. (N) Histology. (O) Masson staining of fibrosis. (P) Immunohistochemistry staining of PRDM16. (Q) Immunohistochemistry staining of p‐p65. (R) Quantification of glomerular damage in kidney samples. (S) Quantification of tubular damage in kidney samples. (T) Quantification of tubulointerstitial fibrosis in kidney samples. (U) Quantification of immunohistochemistry staining of PRDM16. (V) Quantification of immunohistochemistry staining of p‐p65. (W) Immunoblot analysis of PRDM16 and β‐tubulin. (X) Densitometry analysis of immunoblot bands. Original magnification x 400. Scale Bar:100 µm. Data are expressed as mean ± SD (*n* = 6). A&C: #P < 0.05, HG at 24–48 h groups versus NG or mannitol groups. E‐G: # *P < 0.05*, versus NG group * *P < 0.05*, versus HG group. K: #P < 0.05, SC groups versus STZ 4 W or STZ 12 W. M: #P < 0.05, SC 12 W cortex groups versus SC 12 W medulla groups or STZ 12 W cortex groups or STZ 12 W medulla groups. R‐V: #p < 0.05, DKD (early stage) groups or DKD (late stage) groups versus PC groups. X:#p < 0.05, DKD groups versus PC groups.

We first examined PRDM16 expression in high glucose‐incubated renal tubular cells and in STZ‐induced DKD mouse kidneys. In RT–qPCR analysis, PRDM16 mRNA was time‐dependently induced by HG in Boston University mouse kidney proximal tubular (BUMPT) cells, while mannitol had less effects (**Figure** **1**A). Similarly, high glucose (HG) induced PRDM16 protein shown by immunoblot analysis (Figure 1B,C). PRDM16 induction by HG was further confirmed by immunofluorescence staining. In normal glucose (NG) cells, PRDM16 was mainly located in the cytoplasm, but it had nuclear staining as well after HG incubation (Figure 1D). Previous study reported that NF‐ κ B was activated in tubular epithelial cells of DKD.^[^ ^16^ ^]^ We hypothesized that NF‐ κ B might mediate PRDM16 expression during HG treatment. To test this, we examined the effect of TPCA‐1, a commonly used inhibitor of NF‐ κ B signaling. As shown in Figure 1E–G, TPCA‐1 markedly suppressed HG‐induced expression of PRDM16 and NF‐ κ B activation as shown by phosphorylated p65 (p‐p65). According to the JASPAR database predication, we identified 3 potential NF‐ κ B binding sites at the gene promoter of PRDM16, named P1, P2, and P3 (Figure 1H). ChIP analysis verified that HG increased the binding of p65/NF‐ κ B to these sites (Figure 1I). In vivo, C57BL/6J mice were injected with Streptozotocin (STZ) to induce diabetes. The mice after STZ‐treatment had increases in blood glucose, kidney to body weight ratio, and urinary ACR levels than vehicle solution (SC)‐treatment group at 4 weeks, and these changes were further enhanced at 12 weeks after STZ‐treatment (Figure S1A‐S1C). Histology and Masson's trichrome staining showed slight tubular damage, glomerular hypertrophy, and interstitial fibrosis at 4 weeks of STZ treatment, which were further increased at 12 weeks (Figure S1D–H, Supporting Information). Immunoblot analysis showed that PRDM16 expression was noticeably increased at 4 weeks after STZ‐treatment, but this increase was smaller at 12 weeks (Figure 1J,K). Furthermore, immunoblot analysis showed that PRDM16 was markedly induced in both kidney cortex and outer medulla in STZ‐treated mice at 12 weeks after STZ‐treatment (Figure 1L,M). We further examined human DKD renal biopsies with paracancerous (PC) kidney tissues as control. The basic information of the DKD (*n* = 9) and cancer (*n* = 10) patients is presented in Table S1 (Supporting Information). In DKD patients, we further identified 3 at early stage DKD and 3 at late stage DKD according to the severity of renal function (eGFR) decline and proteinuria (Table S4, Supporting Information). Histology and Masson's trichrome staining showed greater tubular damage, glomerular hypertrophy, and interstitial fibrosis in early stage DKD kidney tissues than in PC tissues, these changes were further enhanced in late stage DKD (Figure 1N‐O and 1R–T). Immunohistochemical analyses showed higher levels of PRDM16 and p‐p65/NF‐κB in early stage DKD kidney tissues than in PC tissues, but the increases were reduced in late stage DKD (Figure 1P,Q and 1U,V). Immunoblot analysis confirmed higher PRDM16 expression in DKD kidneys (Figure 1W,X). Together, these data suggest a role of NF‐κ B in disease stage‐dependent expression of PRDM16 in DKD.

### 2.2 PRDM16 Suppresses Fibrotic Protein Expression During HG‐Incubation of BUMPT Cells and in STZ‐Induced Diabetic Mice

**Figure 2:**
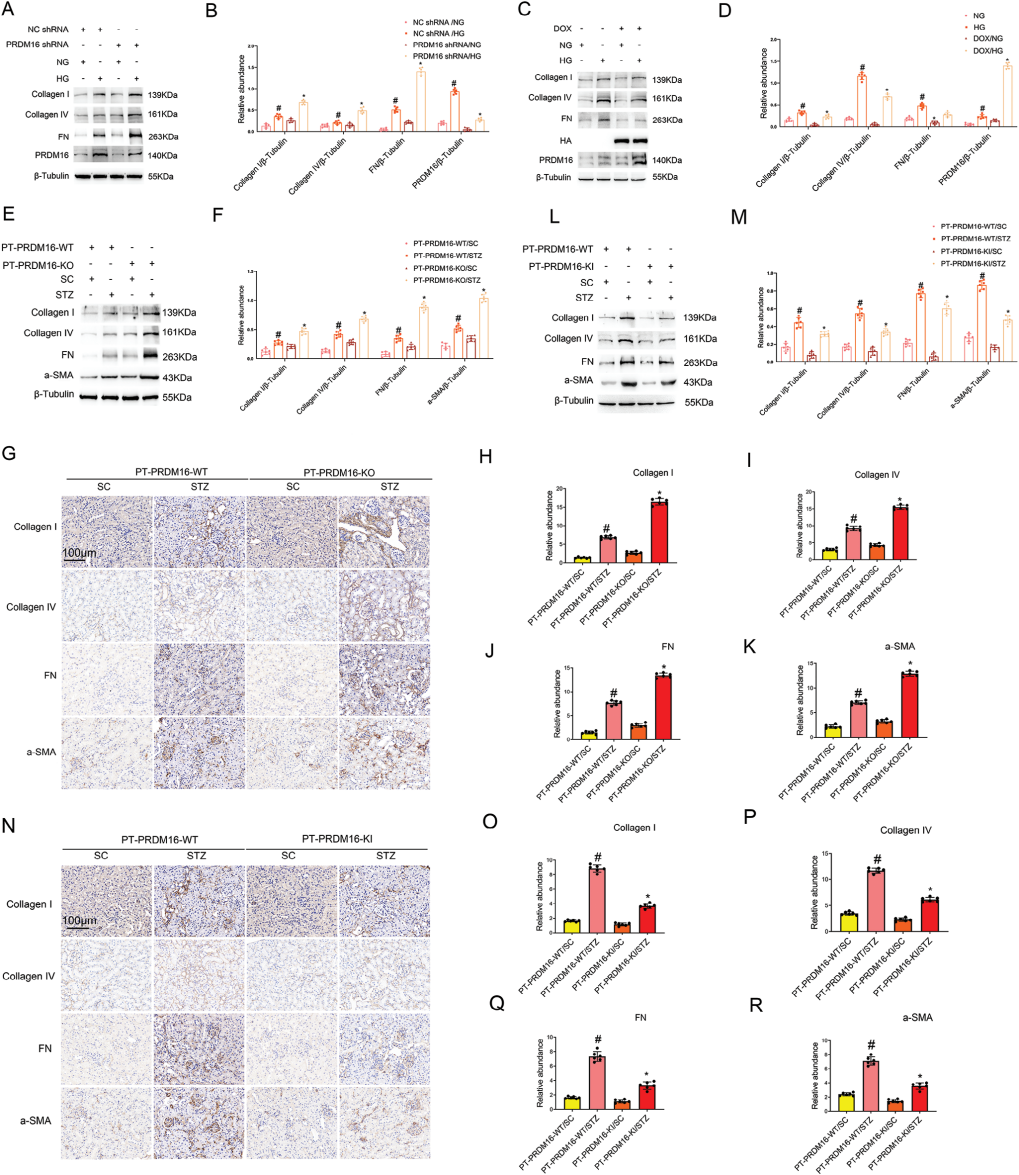
PRDM16 suppresses fibrotic protein expression during HG‐incubation of BUMPT cells and in STZ‐induced diabetic mice. A,B) BUMPT cells were transfected with PRDM16 shRNA or negative control oligos, and then subjected to HG or NG incubation. Cell lysate was collected for immunoblot analysis of Collagen I&IV, FN, PRDM16, and β‐tubulin (**A**), followed by densitometry analysis of immunoblot bands (B). C,D) HA‐PRDM16‐RFP stable BUMPT cells were treated with or without DOX, and then subjected to NG and HG treatment for 48 h. Cell lysate was collected for immunoblot analysis of Collagen I&IV, FN, PRDM16, and β‐tubulin (C), followed by densitometry analysis of immunoblot bands (D). Quantitative data were expressed as mean ± SD (*n* = 6). # *P < 0.05*, versus NC shRNA with NG group. * *P < 0.05*, versus NC shRNA with HG group. E–K) Littermate mice of PT‐PRDM16‐WT and PT‐PRDM16‐KO were intraperitoneally injected with 50 mg kg^−1^ body weight STZ for 5 consecutive days or sodium citrate (SC) as a control. The fasting blood glucose levels of more than 200 mg dL^−1^ for two consecutive measurements were regarded as diabetic, and the mice were kept for another 12 weeks. Tissue protein was collected for immunoblot analysis of Collagen I&IV, FN, a‐SMA, and β‐tubulin (E), followed by densitometry analysis of immunoblot bands (F). Tissue was used for immunohistochemical staining of fibrotic proteins (G), followed by quantification of immunohistochemical staining (H–K). L–R) Littermate mice of PT‐PRDM16‐WT and PT‐PRDM16‐KO were intraperitoneally injected with 50 mg kg^−1^ body weight STZ for 5 consecutive days or sodium citrate (SC) as a control. Tissue protein was collected for immunoblot analysis of Collagen I&IV, FN, a‐SMA, and β‐tubulin(L), followed by densitometry analysis of immunoblot bands (M). Tissue was used for immunohistochemical staining of fibrotic proteins (N), followed by quantification of immunohistochemical staining (O–R). Original magnification x 400. Scale Bar:100 µm. Data are expressed as mean ± SD (n = 6). # *P < 0.05*, versus SC‐treated PT‐PRDM16‐WT group * *P < 0.05*, versus STZ‐treated PT‐PRDM16‐WT group.

To investigate the role of PRDM16, we knocked it down with shRNAs in BUMPT cells and then subjected the cells to NG or HG incubation. The knockdown of PRDM16 noticeably increased the expression of fibrotic proteins, including Collagen I&IV and Fibronectin (Fn) (**Figure** **2**A,B). We further established stable HA‐PRDM16‐RFP‐transfected BUMPT cells that overexpress PRDM16 upon doxycycline (DOX) induction. As shown in Figure 2C,D, DOX‐induced PRDM16 overexpression reduced the expression of fibrotic proteins in cells exposed to HG, further indicating the anti‐fibrotic function of PRDM16. To investigate the role of renal tubule PRDM16, we established a kidney proximal tubule‐specific PRDM16‐knockout (PT‐PRDM16‐KO) mouse model (Figure S2A,B, Supporting Information). Both immunohistochemical and immunoblot analyses proved the lower levels of renal PRDM16 expression in PT‐PRDM16‐KO mice than in PT‐PRDM16‐WT mice (Figure S2C–E, Supporting Information). PT‐PRDM16‐WT and PT‐PRDM16‐KO littermate mice were subjected to STZ induction of diabetes. STZ induced increases in kidney to body weight ratio, blood glucose, and urinary ACR levels (Figure S3A–C, Supporting Information). Compared with wild type mice, PT‐PRDM16‐KO mice had higher kidney to body weight ratio (KW/BW) and urinary albumin/creatinine ratio (ACR) levels at 12 weeks after STZ induction, although they had similar levels of hyperglycemia (Figure S3A–C, Supporting Information). PT‐PRDM16‐KO mice also had more severe tubular and glomerular injury (Figure S3D,F,G, Supporting Information). Masson's trichrome staining showed that PT‐PRDM16‐KO aggravated renal fibrosis in diabetic mice (Figure S3E,H, Supporting Information). Consistently, PT‐PRDM16‐KO kidneys had higher levels of expression of fibrotic proteins including Collagen I&IV, Fibronectin, and a‐SMA (Figure 2E,F,G–K). We further established a proximal tubule‐specific PRDM knock‐in (PT‐PRDM16‐KI) mouse model (Figure S4A–C, Supporting Information). Successful PRDM16 overexpression in renal tubules was confirmed by immunohistochemical and immunoblot analyses (Figure S4D–F, Supporting Information). Male wild‐type (PT‐PRDM16‐WT) and PT‐PRDM16‐KI littermate mice were injected with STZ to induce diabetes and then observed at 12 weeks. PT‐PRDM16‐KI significantly reduced the kidney to body weight ratio or renal hypertrophy, and urinary ACR levels (Figure S5A–C, Supporting Information). In histology, PT‐PRDM16‐KI mice had less tubular and glomerular injury (Figure S5D,F,G, Supporting Information). Masson's trichrome staining indicated that PT‐PRDM16‐KI attenuated renal fibrosis in STZ‐induced diabetic mice (Figure S5E,H, Supporting Information). Consistently, PT‐PRDM16‐KI suppressed fibrotic protein expression (Figure 2L–M,2N–R).

### 2.3 PRDM16 Interacts with the Promoter of TRPA1 to Transactivate its Expression

**Figure 3:**
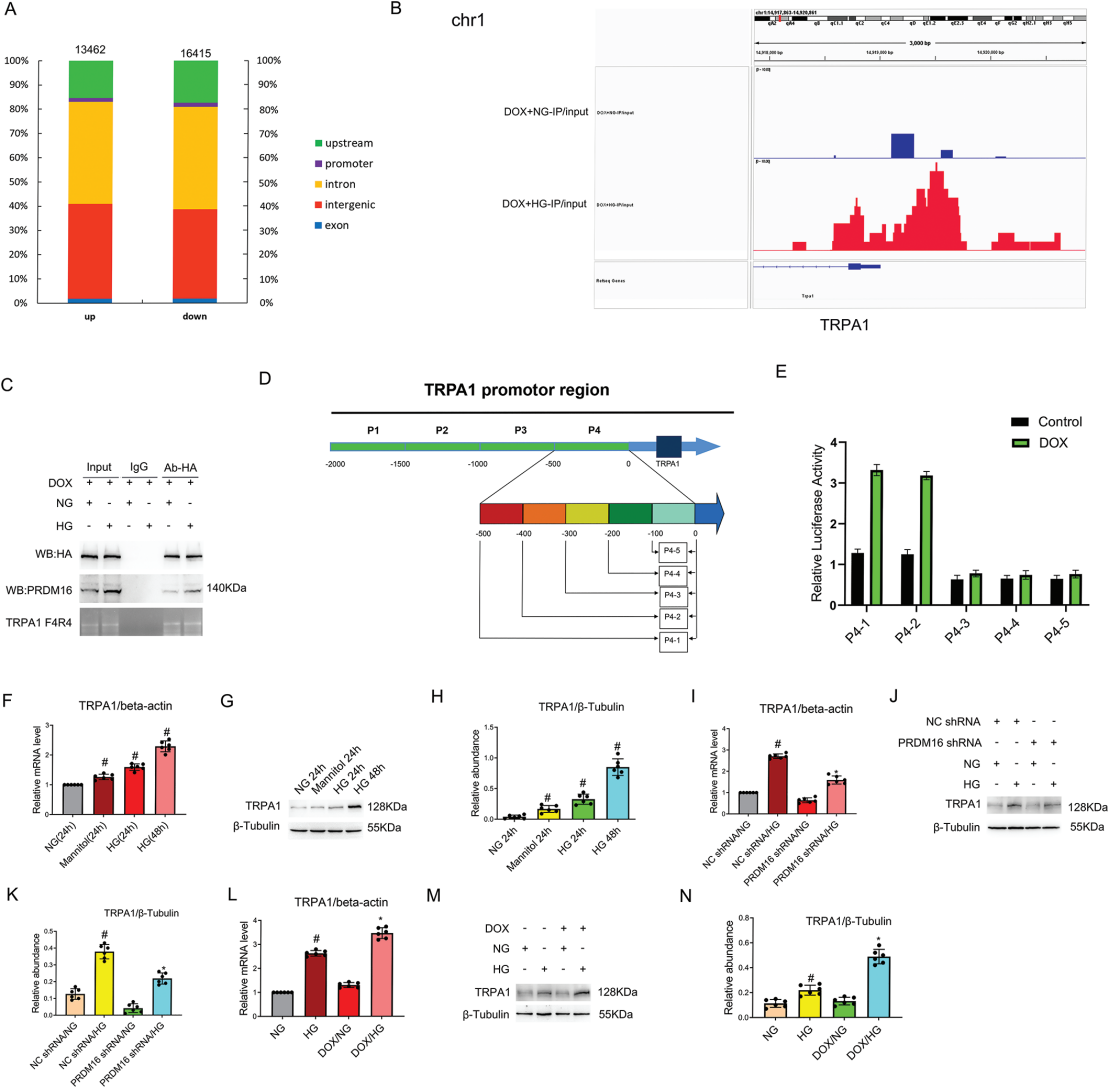
PRDM16 interacts with the promoter of TRPA1 to transactivate its expression. HA‐PRDM16‐RFP stable BUMPT cell line was treated with DOX, and then subjected to NG or HG treatment for 48 h to perform the analysis of ChIP‐seq using HA antibody. A) ChIP‐seq results showing that total 13 462 and 16 415 genes were upregulated and downregulated respectively in HG versus NG group. B) ChIP‐seq image visualized in 10 bp resolution in IGV Genome Browser. C) ChIP assays showing the binding of PRDM16 to the P4 (500‐0 bp) region of TRPA1 gene promoter. D) Map of the promoter region of TRPA1. E) Luciferase reporter assay showing PRDM16 may activate P4‐1 and P4‐2 but no other TRPA1 promoter fragments. F) RT‐qPCR analysis showing HG induction of TRPA1 mRNA. G) Immunoblots showing HG induction of TRPA1 protein. H) Densitometry analysis of immunoblot bands. I–K) Suppression of TRPA1 induction by PRDM16 knockdown in HG‐treated cells. BUMPT cells were transfected with PRDM16 shRNA and then subjected to HG or NG incubation. RT–qPCR and Immunoblot analyses show that knockdown of PRDM16 suppressed the expression of TRPA1. I) RT‐qPCR analysis of TRPA1 mRNA. J) Immunoblot analysis of TRPA1 protein. K) Densitometry analysis of immunoblot bands. L–N) Increased TRPA1 induction by PRDM16 in HG‐treated cells. We established stable HA‐PRDM16‐RFP‐transfected BUMPT cells that overexpress PRDM16 upon doxycycline (DOX) induction. RT–qPCR analysis and Immunoblot results indicates that overexpression of PRDM16 increased the expression of TRPA1.Data are expressed as mean ± SD (*n* = 6). # *P < 0.05*, versus scrambled oligos with NG group. * *P < 0.05*, versus scrambled oligos with HG group.

PRDM16 is a transcription regulator.^[^ ^17^ ^]^ To delineate the anti‐fibrosis mechanism of PRDM16, we analyzed its association with DNA sequences in HG‐incubated cells. HA‐PRDM16‐RFP stable BUMPT cells were treated with DOX to overexpress HA‐tagged PRDM16, and then subjected to NG and HG treatment. We pulled down PRDM16‐associated DNAs with a specific HA antibody for ChIP‐seq assay, which identified 13 462 upregulated and 16 415 downregulated genes in HG cells versus NG group, respectively (**Figure** **3**A). Specifically, PRDM16 bound to the promoter region of 495 genes, among which 218 had increased binding of PRDM16 after HG incubation whereas 277 had decreased binding (Table S2, Supporting Information). Among the upregulated genes, TRPA1 has been implicated in fibrosis. Specifically, TRPA1 was shown to mediate pressure overload‐induced cardiac fibrosis,^[^ ^18^ ^]^ but TRPA1 in T cells suppressed intestinal fibrosis.^[^ ^19^ ^]^ Considering the anti‐fibrotic activity of PRDM16 in renal tubular cells (Figure 2), we hypothesized that it may work by inducing TRPA1. This possibility was supported by the ChIP‐seq image visualized in IGV Genome Browser, which showed an obviously higher enrichment of PRDM16 at TRPA1 promoter in HG cells than in NG cells (Figure 3B). To further test this, we conducted ChIP assay. IP samples were amplified with primers covering different lengths of the TRPA1 gene promoter region (Figure 3D; P1: −2000–1500, P2: −1500–1000, P3: −1000–500, P4: −500‐0). The ChIP assays showed that PRDM16 only interacted with the P4 segment (Figure 3C). Next, the luciferase reporter plasmids containing different lengths of the P4 sequence (P4‐1 to P4‐5 in Figure 3D) were separately transfected into the stable HA‐PRDM16‐RFP‐expressing cell line and then induced with or without DOX for 48 h. The luciferase activity results showed that the promoter activity of P4‐1 and P4‐2 (but not P4‐3, P4‐4, and P4‐5) was significantly increased after DOX induction of PRDM16 overexpression (Figure 3E). Furthermore, TRPA1 was time‐dependently induced by HG in BUMPT cells (Figure 3F–H). Importantly, knockdown of PRDM16 suppressed the expression of TRPA1 at both mRNA and protein levels during HG treatment (Figure 3I–K) whereas overexpression of PRDM16 increased the expression of TRPA1 (Figure 3L–N). These results indicates that PRDM16 interacts with the −400 to −300 bp promoter sequence to induce the expression of TRPA1.

### 2.4 TRPA1 Inhibits the Expression of Fibrotic Proteins during HG‐Incubation of BUMPT Cells and in db/db Diabetic Mice

**Figure 4:**
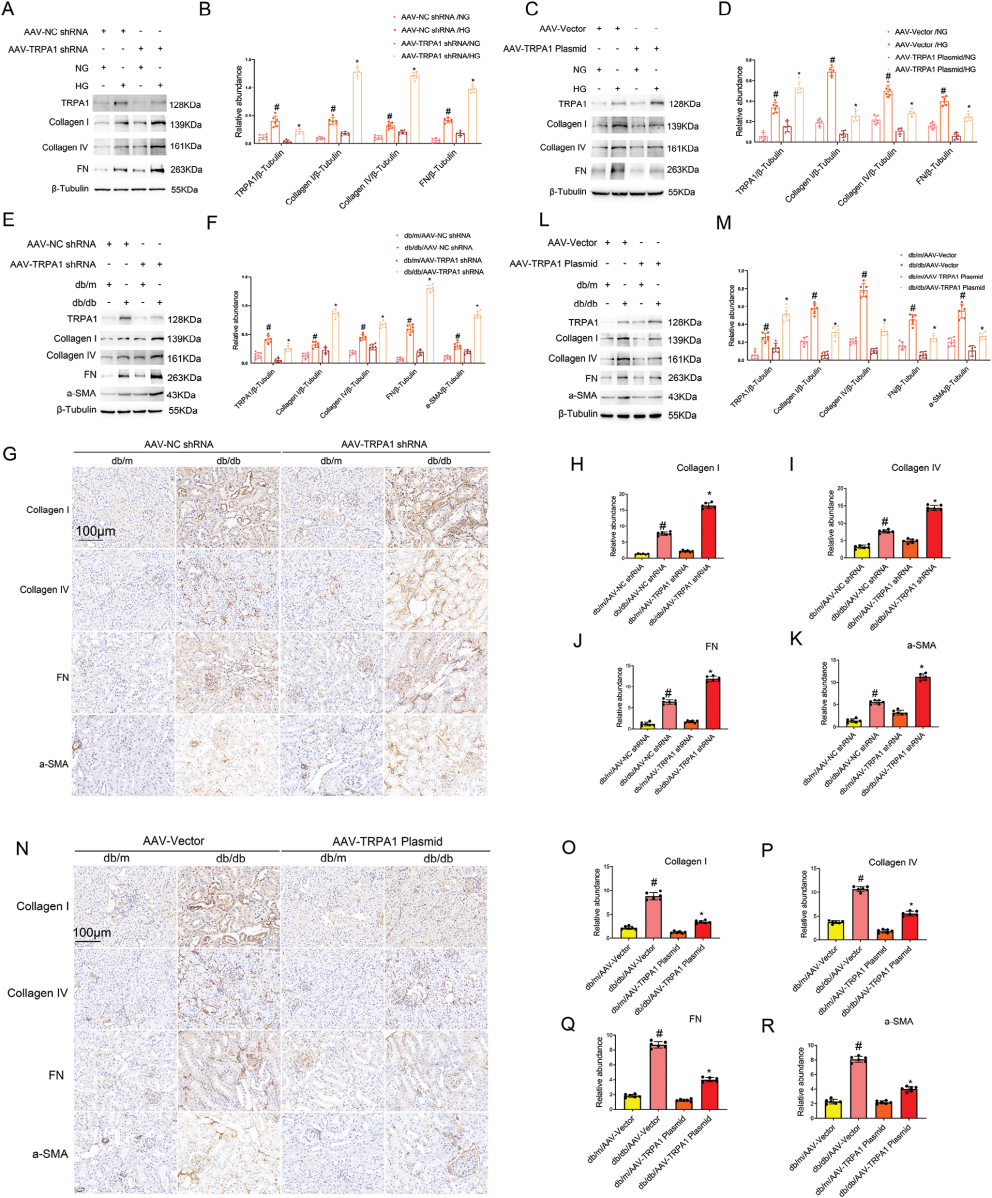
TRPA1 inhibits expression of fibrotic proteins during HG‐incubation of BUMPT cells and in db/db diabetic mice. A,B) BUMPT cells were transfected with AAV2 carrying TRPA1 shRNA or negative control sequence (NC), and then treated with HG for 48 h to collect lysate for immunoblot analysis of indicated proteins. (A) Representative immunoblots. (B) Densitometry analysis of immunoblot bands. C,D) BUMPT cells were transfected with AAV2 vector or AAV2 carrying TRPA1 plasmid, and then treated with HG for 48 h to collect lysate for immunoblot analysis of indicated proteins. (C) Representative immunoblots. (D) Densitometry analysis of immunoblot bands. Quantitative data are expressed as mean ± SD (*n* = 6). # *P < 0.05*, versus scramble with NG group. * *P < 0.05*, versus scramble with HG group. E–K) Eight‐week‐old db/db diabetic mice were injected with AAV2 carrying shRNA or negative control sequence (NC) through vein tail for four weeks. (E) Representative immunoblots. (F) Densitometry analysis of immunoblot bands. (G) Immunohistochemical staining of collagen I&IV, fibronectin, and a‐SMA. (H‐K) Quantification of immunohistochemical staining of them. Original magnification x 400. Scale Bar:100 µm. Data are expressed as mean ± SD (*n* = 6). # *P < 0.05*, versus db/m/NC group. * *P < 0.05*, versus db/db/NC group. L–R) Eight‐week‐old db/db diabetic mice were injected with AAV2 carrying TRPA1 overexpression plasmid or AAV2 empty vector through vein tail for four weeks. (L) Representative immunoblots. (M) Densitometry analysis of immunoblot bands. (N) Immunohistochemical staining of collagen I&IV, fibronectin, and a‐SMA. (O‐R) Quantification of immunohistochemical staining of them. Original magnification x 400. Scale Bar:100 µm. Data are expressed as mean ± SD (*n* = 6). # *P < 0.05*, versus db/m/AAV‐Vector group. * *P < 0.05*, versus db/db/AAV‐Vector group.

To investigate the role of TRPA1 in renal fibrosis, we knocked down TRPA1 in BUMPT cells and then treated the cells with HG for 48 h. Knockdown of TRPA1 increased the expression of fibrotic proteins, including Collagen I&IV and Fibronectin (**Figure** **4**A,B). In contrast, TRPA1 overexpression suppressed the expression of fibrotic proteins (Figure 4C,D). To clarify the role of TRPA1 in vivo, 8‐week‐old db/db diabetic mice were injected with adeno‐associated virus 2 (AAV2) carrying of TRPA1 shRNA via tail vein for four weeks. Knockdown of TRPA1 did not affect hyperglycemia, but it increased kidney to body weight ratio (KW/BW) and urinary albumin/creatinine ratio (ACR) levels (Figure S6A–C, Supporting Information). Histologically, knockdown of TRPA1 increased tubular injury and glomerular damage (Figure S6D,F,G, Supporting Information). Masson's trichrome staining indicated that the knockdown of TRPA1 increased interstitial fibrosis (Figure S6E,H, Supporting Information). Consistently, knockdown of TRPA1 enhanced the expression of Collagen I&IV, Fibronectin, and alpha smooth muscle Actin (a‐SMA) (Figure 4E‐K). We further investigated the effects of TRPA1 overexpression in db/db diabetic mice via AAV2‐TRPA1. As shown in Figure S7A–C (Supporting Information), TRPA1 overexpression reduced the kidney to body weight ratio and urinary ACR changes without affecting blood glucose. In histology, TRPA1 overexpression also attenuated tubular injury, interstitial fibrosis, and glomerular damage (Figure S7D,F,G, Supporting Information). Masson's trichrome staining indicated that TRPA1 overexpression decreased interstitial fibrosis (Figure S7E,H, Supporting Information). Immunoblot and immunohistochemical analyses further showed that TRPA1 overexpression ameliorated the expression of fibrotic proteins, such as Collagen I&IV, Fibronectin, and a‐SMA (Figure 4L–R).

### 2.5 TRPA1 Inhibits MAPK Activation, and TGF‐β1 Expression during HG‐Incubation of BUMPT Cells and in db/db Diabetic Mice

**Figure 5:**
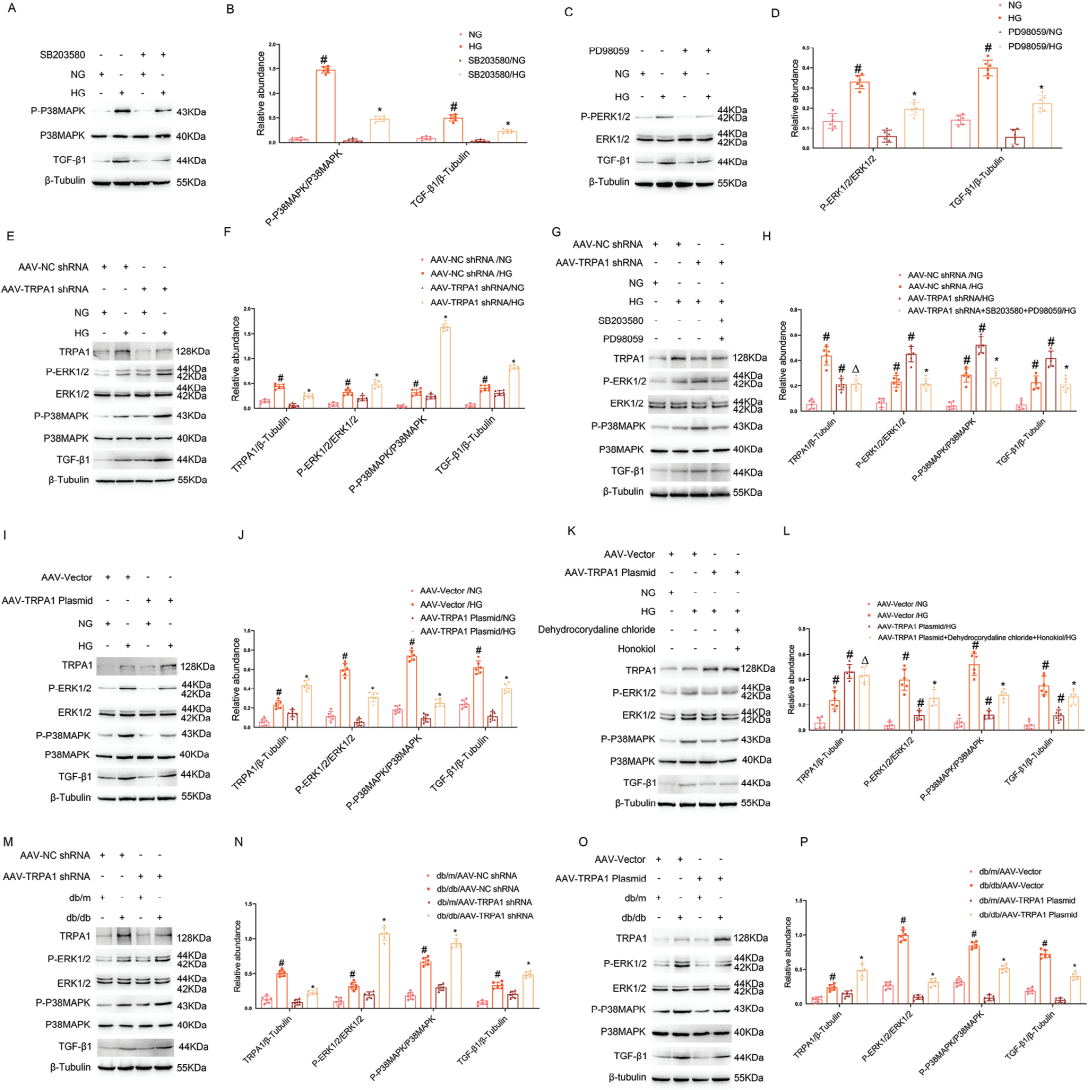
TRPA1 inhibits MAPK activation and TGF‐β1 expression during HG‐incubation of BUMPT cells and in db/db diabetic mice. A–D) BUMPT cells were incubated with normal or high glucose medium for 48 hours in the presence or absence of MAPK inhibitors (10um SB203580 or 10um PD98059) to collect lysate for immunoblot analysis. (A, C) Representative immunoblots. (B, D) Densitometry analysis of immunoblot bands. E,F) BUMPT cells were transfected with AAV2 carrying TRPA1 shRNA or negative control sequence (NC), and then treated with HG for 48 h to collect lysate for immunoblot analysis of indicated proteins. (E) Representative immunoblots. (F) Densitometry analysis of immunoblot bands. Quantitative data are expressed as mean ± SD (*n* = 6). # *P < 0.05*, versus scramble with NG group. * *P < 0.05*, versus scramble with HG group. G,H) BUMPT cells were transfected with AAV2 carrying TRPA1 shRNA or negative control sequence (NC), and then incubated with normal or high glucose medium for 48 h in the presence or absence of MAPK inhibitors (10uM SB203580 or 10uM PD98059). (G) Representative immunoblots. (H) Densitometry analysis of immunoblot bands. Quantitative data are expressed as mean ± SD (*n* = 6). # *P < 0.05*, versus scramble with NG group or HG group. * *P < 0.05* versus scramble with AAV‐TRPA1shRNA with HG group. ^Δ^ *P > 0.05*, versus SB203580 and PD98059 with HG group. I,J) BUMPT cells were transfected with AAV2 vector or AAV2 carrying TRPA1 plasmid, and then treated with HG for 48 h to collect lysate for immunoblot analysis of indicated proteins. (I) Representative immunoblots. (J) Densitometry analysis of immunoblot bands. Quantitative data are expressed as mean ± SD (*n* = 6). # *P < 0.05*, versus scramble with NG group. * *P < 0.05*, versus scramble with HG group. (K‐L) BUMPT cells were transfected with AAV2 vector or AAV2 carrying TRPA1 plasmid, and then incubated with normal or high glucose medium for 48 hours in the presence or absence of MAPK activator (Dehydrocorydaline chloride or Honokiol). K) Representative immunoblots. L) Densitometry analysis of immunoblot bands. Quantitative data are expressed as mean ± SD (*n* = 6). # *P < 0.05*, versus scramble with NG group or HG group. * *P < 0.05* versus scramble with AAV‐TRPA1 Plasmid with HG group. ^Δ^ *P > 0.05*, versus Dehydrocorydaline chloride and Honokiol with HG group. M,N) Eight‐week‐old db/db diabetic mice were injected with AAV2 carrying shRNA or negative control sequence (NC) through vein tail for four weeks. (M) Representative immunoblots. (N) Densitometry analysis of immunoblot bands. Data are expressed as mean ± SD (*n* = 6). # *P < 0.05*, versus db/m/NC group. * *P < 0.05*, versus db/db/NC group. O,P) Eight‐week‐old db/db diabetic mice were injected with AAV2 carrying TRPA1 overexpression plasmid or AAV2 empty vector through vein tail for four weeks. (O) Representative immunoblots. (P) Densitometry analysis of immunoblot bands. Data are expressed as mean ± SD (*n* = 6). # *P < 0.05*, versus db/m/AAV‐Vector group. * *P < 0.05*, versus db/db/AAV‐Vector group.

In BUMPT cells, HG induced the expression of TGF‐β1 and, notably, this induction was blocked by MAPK inhibitors (SB203580 for P38 inhibitor and PD98059 for ERK1/2 inhibitor), indicating the MAPK/TGF‐β1 signaling axis of fibrosis (**Figure** **5**A–D). Mechanistically, knockdown of TRPA1 led to P38 and ERK1/2 MAPK activation accompanied by TGF‐β1 induction (Figure 5E,F), which was diminished by MAPK inhibitors (Figure 5G,H). In contrast, TRPA1 overexpression suppressed the MAPK activation and TGF‐β1 induction (Figure 5I,J), and this effect was mostly recovered by MAPK activators (Dehydrocorydaline chloride for P38 activator and Honokiol for ERK1/2 activator) (Figure 5K–L). These results indicate that TRPA1 may suppress HG‐induced renal fibrosis by blocking the MAPK/TGF‐β1 signaling axis. To confirm the in vitro finding that TRPA1 suppresses the MAPK/TGF‐β1 signaling axis, their expression was examined in db/db diabetic mice. Knockdown of TRPA1 noticeably increased the activation of P38MAPK and ERK1/2 and expression of TGF‐β1 in db/db diabetic mice kidneys (Figure 5M,N). In addition, the activation of P38 and ERK1/2 MAPKs and the induction of TGF‐β1 were suppressed by TRPA1 overexpression (Figure 5O,P). The loss and gain of function studies in Figures 4 and 5 indicate that TRPA1 may protect against the development of tubulo‐interstitial pathologies in DKD by blocking the MAPK/TGF‐β1 pathway.

### 2.6 PRDM16 Suppresses HG‐Induced and STZ‐Induced MAPK Activation and TGF‐β1 Expression in Renal Tubular Cells Upstream of TRPA1

**Figure 6:**
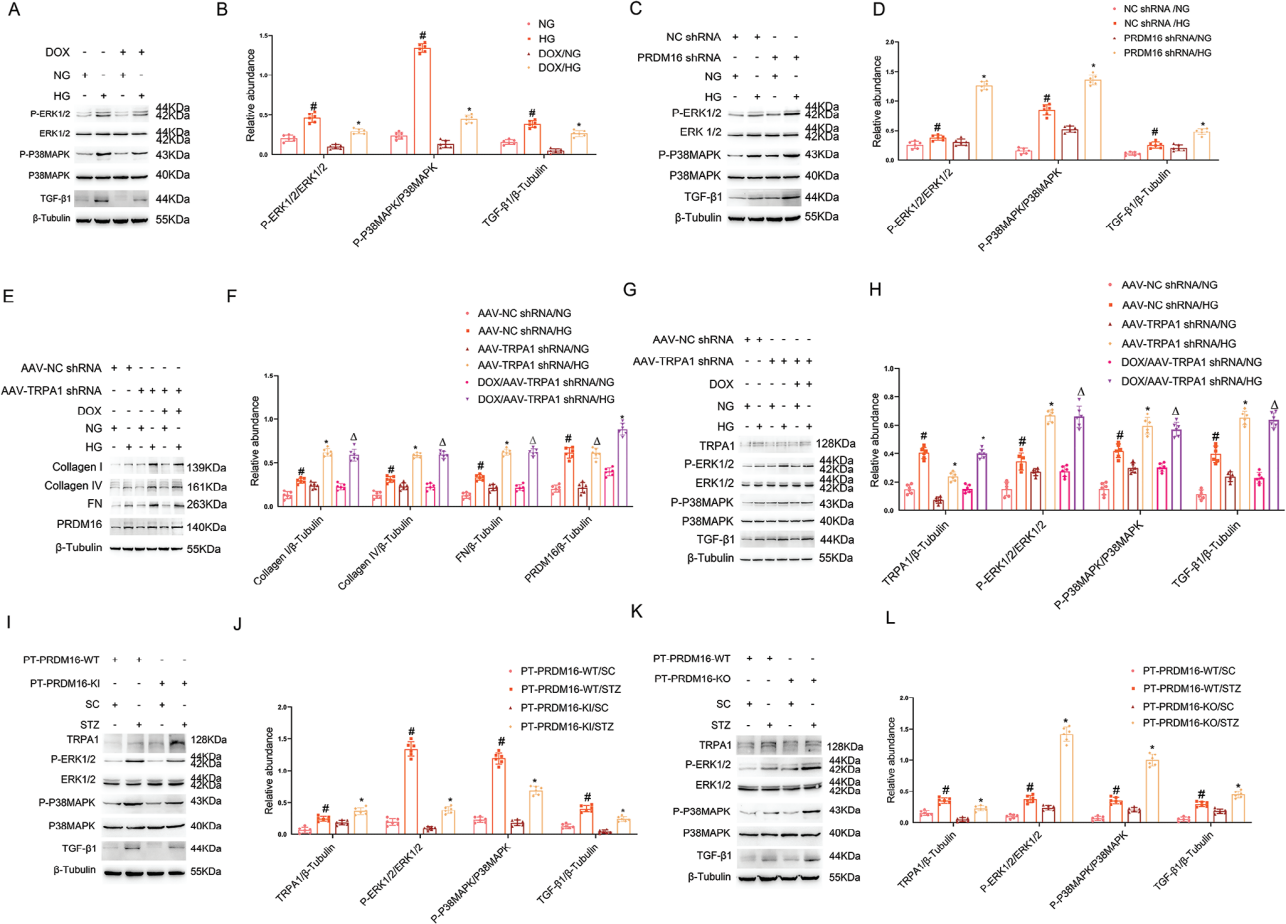
PRDM16 suppresses HG‐induced and STZ‐induced MAPK activation and TGF‐β1 expression in renal tubular cells upstream of TRPA1. A,B) PRDM16‐RFP stably transfected cells with or without DOX induction were treated with NG or HG 48 h to collect lysate. (A) Immunoblot analysis showing the inhibitory effect of PRDM16 on MAPK activation and TGF‐β1 expression. (B) Densitometry analysis of immunoblot bands. C,D) PRDM16 was knocked down with shRNAs in BUMPT cells and then subjected the cells to HG or NG incubation. (C) Immunoblot analysis showing the enhancing effect of PRDM16 knockdown on MAPK activation and TGF‐β1 expression. (D) Densitometry analysis of immunoblot bands. Data are expressed as mean ± SD (*n* = 6). # *P < 0.05*, versus scramble with NG group. * *P < 0.05*, versus scramble with HG group. E,F) Cells stably expressing HA‐PRDM16‐RFP were transfected with the AAV2 carrying TRPA1 shRNA, and then treated with NG or HG with or without DOX induction for 48 h. (E) Immunoblot analysis of fibrotic proteins. (F) Densitometry analysis of immunoblot bands. (G) Immunoblot analysis of MAPK activation and TGF‐β1 expression. (H) Densitometry analysis of immunoblot bands. Data are expressed as mean ± SD (*n* = 6). # *P < 0.05*, versus scramble with NG group. * *P < 0.05* versus scramble with HG group. ^Δ^ *P > 0.05*, versus TRPA1 with NG or HG group. I–L) Littermate mice of PT‐PRDM16‐WT and PT‐PRDM16‐KI or PT‐PRDM16‐KO were intraperitoneally injected with 50 mg kg^−1^ body weight STZ for 5 consecutive days or sodium citrate (SC) as a control. The fasting blood glucose levels of more than 200 mg dL^−1^ for two consecutive measurements were regarded as diabetic, and the mice were raised for another 12 weeks. I) Immunoblot analysis showing the inhibitory effect of PRDM16 on MAPK activation and TGF‐β1 expression. J) Densitometry analysis of immunoblot bands. K) Immunoblot analysis showing the enhancing effect of PRDM16 knockdown on MAPK activation and TGF‐β1 expression. L) Densitometry analysis of immunoblot bands. Data are expressed as mean ± SD (*n* = 6). # *P < 0.05*, versus SC‐treated PT‐PRDM16‐WT group * *P < 0.05*, versus STZ‐treated PT‐PRDM16‐WT group.

We have presented the evidence that PRDM16 transactivates TRPA1 expression (Figure 3), and TRPA1 suppresses MAPK activation and TGF‐β1 expression in DKD (Figures 5 and 6). Hence, we hypothesized that PRDM16 might suppress MAPK activation and TGF‐β1 expression. Indeed, PRDM16 overexpression inhibited the activation of P38 and ERK1/2, accompanied by less TGF‐β1 expression during HG incubation of BUMPT cells (**Figure** **6**A,B), whereas knockdown of PRDM16 had opposite effects (Figure 6C,D). We further determined whether TRPA1 acts downstream of PRDM16 for suppressing renal fibrosis. TRPA1 was knocked down in PRDM16‐overexpressing cells that were then subjected to HG incubation. Immunoblot analysis indicated that TRPA1 knockdown markedly enhanced fibrotic protein expression, and these changes were not attenuated by PRDM16 overexpression (Figure 6E,F). Furthermore, PRDM16 overexpression did not ameliorate MAPK activation and TGF‐β1 induction in TRPA1‐knockdown cells during HG incubation (Figure 6G,H). Collectively, these data indicate that TRPA1 is a downstream mediator of the inhibitory effects of PRDM16 on renal fibrosis. To confirm the in vitro finding that PRDM16 promotes the TRPA1 to block the MAPK/TGF‐β1 axes. We examined their expression in PRDM16‐KO or KI mice treated with or without STZ treatment. The immunoblot results showed that PT‐PRDM16‐KO kidney tissues had less TRPA1 but higher MAPK activation and TGF‐β1 induction (Figure 6I,J). However, PT‐PRDM16‐KI increased TRPA1 and suppressed MAPK activation and TGF‐β1 expression (Figure 6K,L). Collectively, these data indicate that PRDM16 in renal proximal tubules has a protective role against renal tubulo‐interstitial pathologies in DKD by inducing TRPA1 to suppress the MAPK/TGF‐β1 axis.

### 2.7 PRDM16‐Knockdown BUMPT Cells Secretes more TGF‐β 1 to Induce Fibrotic Protein Production in Fibroblasts

Tubular cells may secrete profibrotic factors to stimulate interstitial fibroblasts for renal fibrosis.^[^ ^20^, ^21^, ^22^, ^23^, ^24^ ^]^ Therefore, we tested if PRDM16 in renal tubular cells might suppress pro‐fibrotic factor production to inhibit fibroblast activation. To test this, PRDM16‐knockdown BUMPT cells and control cells were cultured for 24 h to collect their media, which were separately added to NIH3T3 fibroblasts. To determine the involvement of TGF‐β1, we tested the effects of TGF‐β1 neutralizing antibodies (Ab‐TGF‐β1) (Figure S8A, Supporting Information). The medium from PRDM16‐knckdown BUMPT cells induced the expression of Fibronectin, Collagens, and a‐SMA in fibroblasts, which was noticeably inhibited by TGF‐β1 neutralizing antibodies (Figure S8B,C, Supporting Information). Compared with negative control shRNA‐transfected cells, PRDM16‐knockdown BUMPT cells secreted higher levels of TGF‐β1 into culture medium, which were neutralized by Ab‐TGF‐β1 (Figure S8D, Supporting Information). These data suggest that PRDM16 in renal tubular cells may suppress renal fibrosis by blocking TGF‐β1 production and its paracrine action on interstitial fibroblasts.

### 2.8 ADV‐Mediated PRDM16 Overexpression Attenuates Renal Fibrosis, MAPK Activation, and TGF‐β1 Expression in db/db Diabetic Mice

**Figure 7:**
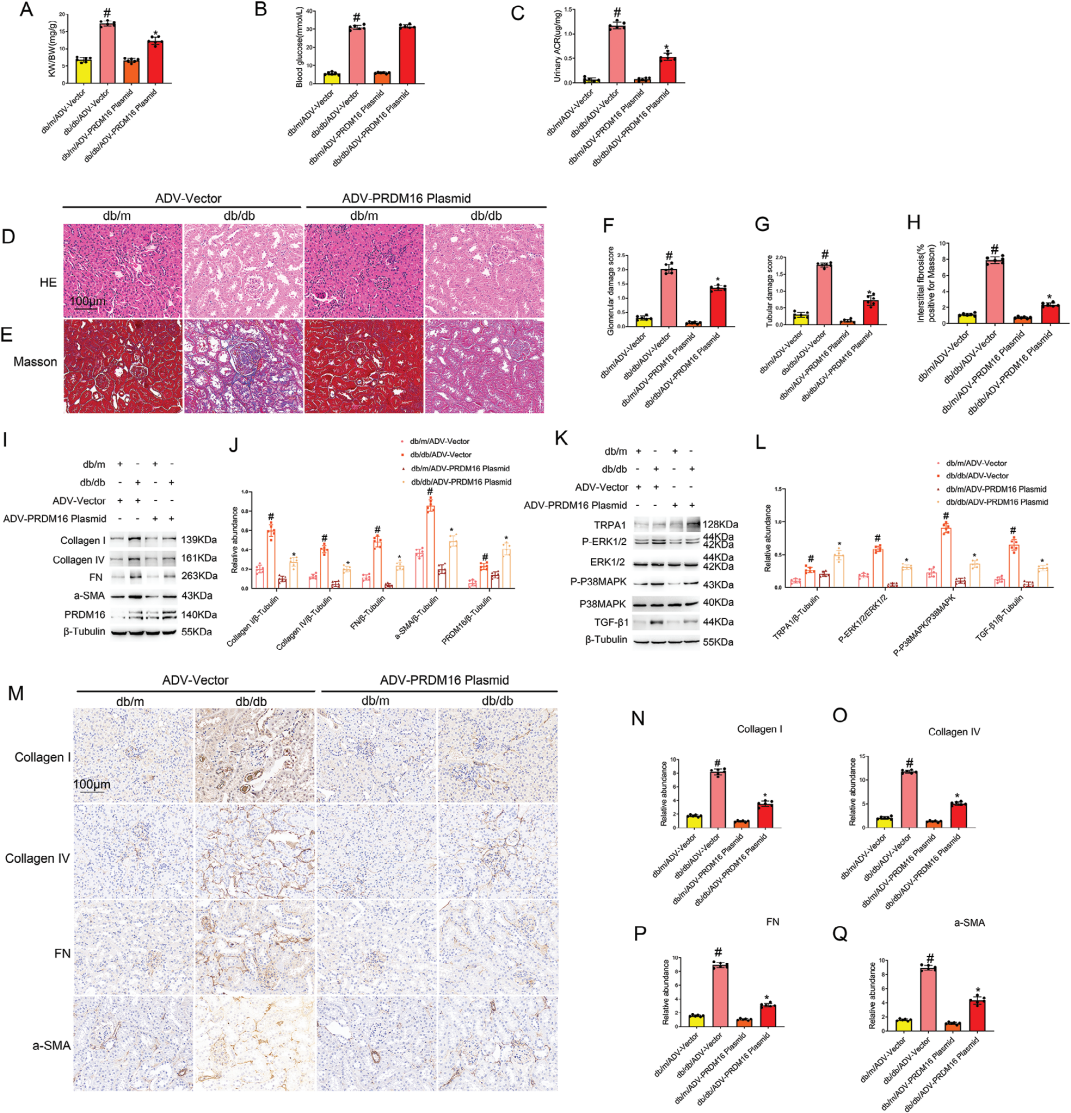
ADV‐mediated PRDM16 overexpression attenuates renal fibrosis, MAPK activation, and TGF‐β1 expression in db/db diabetic mice. Eight‐week‐old db/db diabetic mice were injected with ADV carrying TRPA1 overexpression plasmid or ADV empty vector through tail vein for 4 weeks. A) KW/BW. B) Fasting blood glucose. C) Urinary ACR. D) Representative images of H&E staining. E) Representative images of Masson staining. F) Quantification of glomerular damage score. G) Quantification of tubular damage score. H) Quantification of tubulointerstitial fibrosis in the kidney cortex. I,K) Representative immunoblots. J,L) Densitometry analysis of immunoblot bands. M) Immunohistochemical staining analysis of collagen I&IV, fibronectin, and a‐SMA. N–Q) Quantification of immunohistochemical staining of them. Original magnification x 400. Scale Bar:100 µm. Data are expressed as mean ± SD (*n* = 6). # *P < 0.05*, versus db/m/ADV‐Vector group. * *P < 0.05*, versus db/db/ADV‐Vector group.

We further investigated the effects of adenovirus (ADV)‐mediated PRDM16 overexpression plasmids in db/db diabetic mice. As shown in **Figure** **7**A–C, PRDM16 overexpression markedly attenuated the increases in kidney to body weight ratio, and urinary ACR levels in db/db diabetic mice. HE staining showed that PRDM16 suppressed tubular epidermal destruction and glomerular hypertrophy in these mice (Figure 7D), which was confirmed by the quantification of glomerular and tubular damage (Figure 7F,G). Masson's trichrome staining showed that PRDM16 attenuated renal fibrosis in db/db diabetic mice (Figure 7E,H). Immunoblot and immunohistochemical analyses further showed that PRDM16 overexpression ameliorated the expression of fibrotic proteins, such as Collagen I&IV, Fibronectin, and a‐SMA (Figure 7I,J and 7M–Q). In addition, PRDM16 blocked the activation of MAPK/TGF‐β1 pathway by upregulating TRPA1 (Figure 7K,L).

### 2.9 Formononetin Attenuates Renal Fibrosis, MAPK Activation, and TGF‐β1 Expression in db/db Diabetic Mice

**Figure 8:**
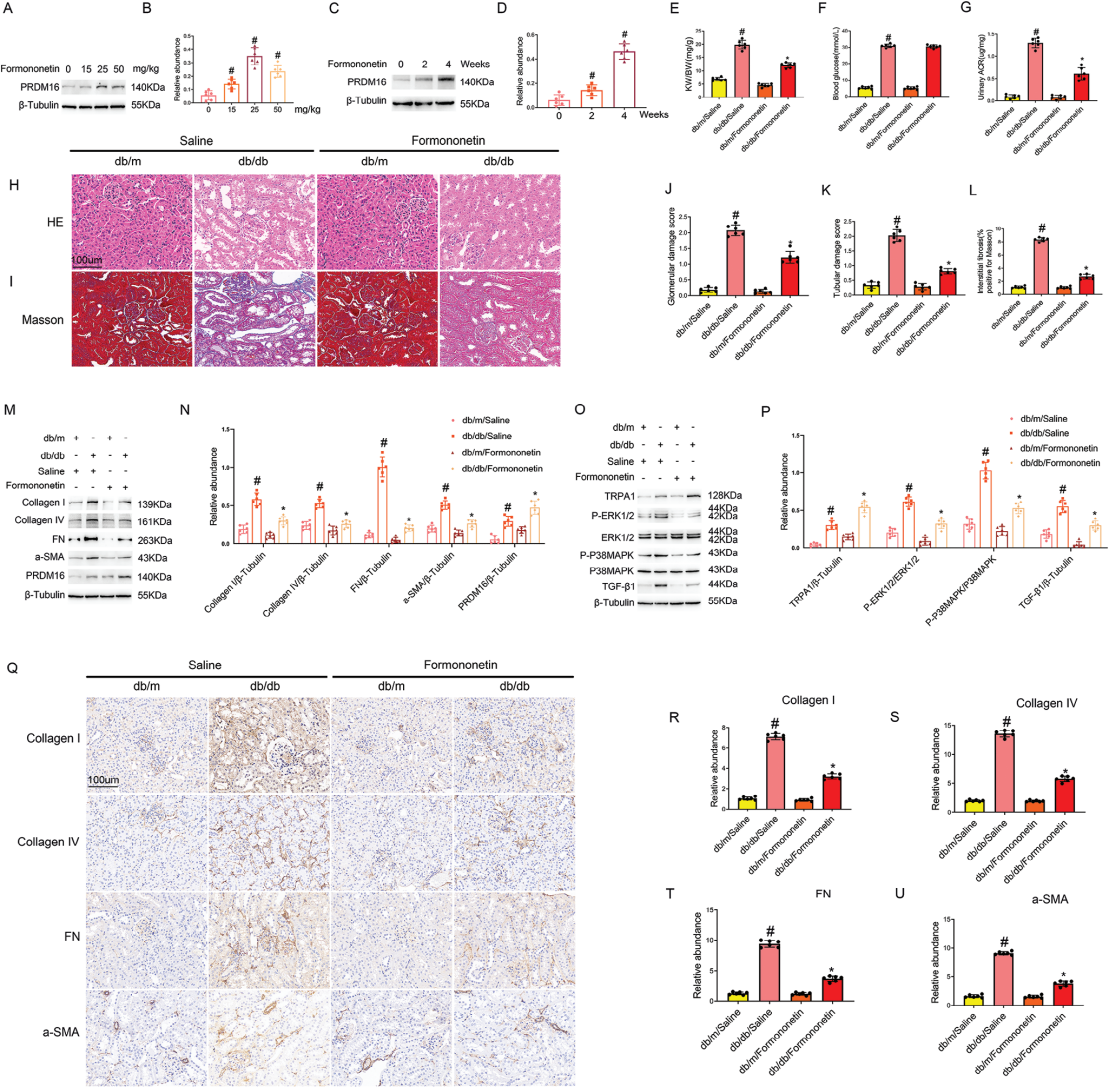
Formononetin attenuates renal fibrosis, MAPK activation, and TGF‐β1 expression in db/db diabetic mice. Eight‐week‐old db/db diabetic mice were intragastrically administered formononetin at a dose of 25 mg kg^−1^ once a day for four weeks to collect samples for analysis. A,C) Representative immunoblots. B,D) Densitometry analysis of immunoblot bands. E) kidney to body weight ratio (KW/BW). F) Fasting blood glucose. G) Urinary ACR. H) Representative images of H&E staining. I) Representative images of Masson staining. J) Quantification of glomerular damage score. K) Quantification of tubular damage score. L) Quantification of tubulointerstitial fibrosis in the kidney cortex. M,O) Representative immunoblots. N,P) Densitometry analysis of immunoblot bands. Q) Immunohistochemical staining of collagen I&IV, fibronectin, and a‐SMA. R–U) Quantification of immunohistochemical staining of them. Original magnification x 400. Scale Bar:100 µm. Data are expressed as mean ± SD (*n* = 6). # *P < 0.05*, versus db/m/Saline group. * *P < 0.05*, versus db/db/Saline group.

Formononetin is a type of phytoestrogen isolated from the Chinese medicine herb Red Clover, which has been reported to attenuate diabetic renal fibrosis,^[^ ^25^, ^26^ ^]^ but the underlying mechanism remains unclear. We treated db/db diabetic mice with formononetin at doses of 0, 15, 25, and 50 mg kg^−1^ for 3 days. Immunoblotting demonstrated that the expression of PRDM16 was induced by 15 mg kg^−1^ formononetin, peaked at 25 mg kg^−1^, and then gradually declined at 50 mg kg^−1^ (**Figure** **8**A,B), indicating formononetin is an inducer or activator of PRDM16. We then chose 25 mg kg^−1^ formononetin for 0, 2, and 4 weeks of treatment. Immunoblotting showed a treatment time‐dependent induction of PRDM16 by formononetin (Figure 8C,D). To clarify whether formononetin may directly bind to PRDM16, we conducted a molecular docking analysis, which indicated that formononetin binds to PRDM16 with a high affinity of −5.2 kcal mol^−1^ and it targets the drug binding pocket of PRDM16 surrounded by amino acids such as R355, S359, F376, and T378 (Figure S11, Supporting Information). Functionally, 4 weeks of formononetin reduced the kidney to body weight ratio and urinary ACR in db/db diabetic mice without affecting blood glucose (Figure 8E–G). In histology, formononetin reduced tubular and glomerular injury (Figure S8H,J,K, Supporting Information). Masson's trichrome staining indicated that formononetin attenuated renal fibrosis in db/db diabetic mice (Figure 8I,L). Immunoblot and immunohistochemical analyses further showed that formononetin ameliorated the expression of fibrotic proteins, such as Collagen I&IV, Fibronectin, and a‐SMA (Figure 8M,N, 8Q–U). In addition, the activation of MAPKs and the induction of TGF‐β1 were suppressed by formononetin (Figure 8O,P). These results indicate that formononetin may induce PRDM16 to protect kidneys against DKD.

### 2.10 PRDM16/TRPA1/MAPK/TGF‐β1 Axis in DKD Patients

Human DKD renal biopsies had elevated expression of PRDM16 (Figure 1). We further examined TRPA1, P38 and ERK1/2, TGF‐β1, and fibrotic proteins in kidney tissues of DKD. Histology and Masson's trichrome staining showed greater tubular injury, glomerular damage (Figure 1), and interstitial fibrosis in DKD renal biopsies than in para‐cancer normal kidney tissues (PC). DKD samples also exhibited higher expression of collagen I&IV, a‐SMA, and fibronectin in immunohistochemical and immunoblot analyses (Figure S9A–G, Supporting Information). TRPA1 was also higher in DKD kidney tissues by immunoblot analysis. Moreover, DKD samples had higher levels of phosphorylation/activation of P38 and ERK1/2, associated with TGF‐β1 induction (Figure S9H,I, Supporting Information). These analyses verify the activation of the PRDM16/TRPA1/MAPK/TGF‐β1 pathway for renal tubulo‐interstitial pathologies in human DKD.

## 3 Discussion

Compared with other forms of CKD, DKD has unique features.^[^ ^2^, ^3^, ^4^ ^]^ Especially, at the pathological level it is surprising that renal tubulo‐interstitial fibrosis (TIF), a common characteristic of CKD, does not develop significantly until the late stage of DKD.^[^ ^2^, ^3^, ^4^, ^7^ ^]^ In the current study, we demonstrate that PRDM16 is up‐regulated in renal tubular cells at the early stage of DKD and plays an important role in suppressing TIF development. Mechanistically, we show that PRDM16 transcriptionally activates TRPA1, which then suppresses P38 and ERK1/2 MAPKs to block TGF‐β1 production in renal tubular cells (Figure S12, Supporting Information). Of note, we have examined DKD models of both type 1 and type 2 diabetes and have verified the molecular changes in human DKD kidney samples. Collectively, these findings provide new insights into the regulation of renal fibrosis in DKD and the pathogenesis of DKD in general.

PRDM16 was originally identified through the aberrant expression of its short variant in acute myeloid leukemia.^[^ ^8^ ^]^ However, the majority of recent studies linking PRDM16 to disease is in the context of adipose tissue metabolism, including browning and thermogenesis of adipocytes and the beigeing of adipocytes.^[^ ^15^, ^27^, ^28^ ^]^ Closely relevant to our present work, two studies showed that PRDM16 was anti‐fibrotic in adipose tissues.^[^ ^14^, ^15^ ^]^ Cibi et al. further reported that PRDM16 suppressed cardiac hypertrophy and ventricular fibrosis associated with aging.^[^ ^29^ ^]^ PRDM16 is expressed in kidneys, but nothing is known about its involvement in renal homeostasis and disease. In this study, we show that NF‐ κ B mediates the upregulation of PRDM16, which suppresses renal fibrosis in early DKD. Especially, knockout of PRDM16 from kidney tubule cells increased TIF in early DKD mice (Figure 2), whereas overexpression of PRDM16 in knock‐in mice diminished it (Figure 2). These results support an anti‐fibrosis function of PRDM16 in kidneys.

PRDM16 is a member of the PRDM protein family that contain an N‐terminal PRDI‐BF1 and RIZ1 homology domain and two clusters of zinc fingers. Functionally, PRDM16 has intrinsic histone methyltransferase activity and may therefore regulate gene transcription by modifying chromatin via histone methylation. In addition, it may recruit cofactors and other chromatin modifiers.^[^ ^17^ ^]^ In adipose tissue, PRDM16 was shown to interact with general transcription factor 2I repeat domain‐containing protein 1 (GTF2IRD1) to suppress the expression of fibrosis genes.^[^ ^14^ ^]^ PRDM16 may also promote the secretion of the metabolite β‐hydroxybutyrate in adipose cells, which acts as a paracrine factor to suppress myofibroblast formation and, in turn, fibrogenesis.^[^ ^15^ ^]^ In our study, ChIP‐seq analysis suggested that PRDM16 bound to the sequences of quite a few genes in high glucose or diabetic condition. Further analysis, including luciferase reporter assays, verified that PRDM16 transactivates TRPA1 by directly binding to its gene promotor. We narrowed down the binding region to 100 bp in TRPA1 gene promoter (Figure 3), but further work needs to pinpoint the core binding sequence.

TRPA1 is a nonselective cation channel protein that has been implicated in the progression of fibrosis, but its role is cell type and context‐dependent.^[^ ^18^, ^19^, ^30^ ^]^ In mice, pharmacological inhibition of TRPA1 ameliorated pressure overload‐induced cardiac hypertrophy and fibrosis,^[^ ^19^ ^]^ suggesting a pro‐fibrotic role in TRPA1. On the contrary, knockout of TRPA1 increased age‐related cardiac fibrosis, intestinal fibrosis in experimental Crohn's disease, and bleomycin‐induced dermal fibrosis.^[^ ^19^, ^31^, ^32^ ^]^ Our present study reports the first evidence for the involvement of TRPA1 in renal fibrosis. Especially, knockdown of TRPA1 enhanced TGF‐β1 expression and renal interstitial fibrosis in db/db diabetic mice, whereas overexpression of TRPA1 had opposite effects (Figures 4 and 5). These and other results demonstrate that TRPA1 is anti‐fibrotic in early DKD. Mechanistically, we have further collected evidence that TRPA1 may suppress renal fibrosis by blocking MAPK (P38 and ERK1/2) activation and consequent TGF‐β1 expression (Figures 4 and 5). Our observation of the involvement of P38 and ERK1/2 signaling in DKD is consistent with previous reports.^[^ ^33^, ^34^ ^]^ In addition, previous studies reported that P38 and ERK1/2 signaling mediated TGF‐β1 expression during high glucose incubation of bone marrow stem cells and peritoneal mesothelial cell.^[^ ^35^, ^36^ ^]^ However, our observation of negative regulation of MAPKs by TRPA1 is at discrepancy with published work. For example, Okada et al reported that TRPA1 enhanced the TGF‐β‐induced the activation of P38 and ERK1/2 in ocular fibroblasts.^[^ ^37^ ^]^ Soni et al. also demonstrated that TRPA1 mediated the interleukin 1 beta‐induced mesangial proliferation via activation of ERK1/2.^[^ ^38^ ^]^ Apparently, whether TRPA1 inhibits or activates MAPKs may depends on the cell types and stimuli. In DKD, our work suggests that TRPA1 is induced via PRDM16 and, upon induction, TRPA1 may suppress MAPK and associated TGF‐β1 expression in renal tubule cells.

TGF‐β signaling is a major driving force in renal fibrosis.^[^ ^39^ ^]^ Upon ligand binding, TGF‐β receptors are activated and phosphorylate downstream signal transducers, especially Smad proteins, which accumulate in the nucleus to transactivate a variety of pro‐fibrotic genes.^[^ ^39^ ^]^ There are scattered reports about the connection between PRDM16 and TGF‐β signaling. In 2009, Takahata et al. showed that PRDM16 may associate with the Smad complex to repress TGF‐β‐associated gene transcription. The latest work by Hurwitz et al. further proves the antagonism between PDRM16 and Smad4, where Smad4 interacts with and recruits PDRM16 to suppress its own expression.^[^ ^40^ ^]^ These studies indicate an interesting reciprocal regulation between PRDM16 and TGF‐β signaling through direct interactions with Smad proteins. In the present study, we demonstrate that PRDM16 may antagonize TGF‐β signaling by inducing TRPA1 to suppress MAPK‐mediated TGF‐β1 expression. This finding unveils a new mechanism whereby PDRM16 represses TGF‐β signaling indirectly via TRPA1. In addition, the culture medium transfer experiment showed that tubular PRDM16 suppressed the activation of fibroblasts by reducing the secretion of TGF‐β1 (Figure S8, Supporting Information). Recent studies reported that formononetin attenuates diabetic renal fibrosis by activating the Nrf2/ARE pathway and inactivating the Smad3 pathway, respectively.^[^ ^25^, ^26^ ^]^ In our study, we for the first time demonstrated that formononetin could directly bind to PRDM16 and upregulate PRDM16 to promote the expression of TRPA1 to suppress the MAPK/TGF‐β1 axes of renal fibrosis in db/db diabetic mice (Figure 8; Figure S11, Supporting Information).

In summary, we have demonstrated the first evidence that PRDM16 contributes to the low level of TIF in the early stage of DKD. Mechanistically, PRDM16 transcriptionally transactivates TRPA1 expression and, upon induction, TRPA1 inhibits P38 and ERK1/2 MAPKs to suppress TGF‐β1 expression and associated fibrosis. Taken together, our data showed that PRDM16 exerts an antifibrotic effect and may serve as a novel therapeutic target for DKD. Together, these findings unveil a new anti‐fibrotic pathway, involving PRDM16, TRPA1, MAPK and TGF‐β1, that is activated in renal tubule cells in early DKD to keep fibrosis at a low level.

## 4 Experimental Section

### Animal Models

C57BL/6J male mice were obtained from Hunan SJA Laboratory Animal Company. The C57BL/6J KsJ‐leprdb (db/db) and WT control C57 (+/+) mice were purchased from Shanghai Animal Center (Shanghai, China). All mice were maintained in a specific pathogen‐free facility at 22–25°C under a light–dark cycle with free access to a standard rodent diet and water. All animal experiments complied with the guideline approved by the Animal Care Ethics Committee of Second Xiangya Hospital, China (NO. 2 018 065).

### PT‐PRDM16‐KO Mice

The mouse model of proximal tubule PRDM16 KO was established to further investigate the role of PRDM16 in vivo. Briefly, the floxed PRDM16 (obtained from The Jackson Laboratory,USA) alleles in male mice (PRDM16 ^f/f^ X^cre^Y) were crossed with female phosphoenolpyruvatecarboxy kinase‐cAMP‐response element (PEPCK‐Cre) (provided by Volker Haase of Vanderbilt University School of Medicine, Nashville, TN) transgenic mice (PRDM16^+/+^X^cre^X^cre^) to produce heterozygous female offspring (PRDM16^f/+^X^cre^X) and then crossed with PRDM16 ^f/f^XY males to produce littermate proximal tubule PRDM16 wild‐type (PT‐PRDM16‐WT) and PT‐PRDM16‐KO (PRDM16^f/f^X^cre^Y) mice (Figure S2A, Supporting Information). Each mouse was subjected to three sets of PCR test. The genotype of PT‐PRDM16‐KO mice was characterized by^[^ ^1^ ^]^ amplification of the 187‐bp DNA fragment for the floxed allele,^[^ ^2^ ^]^ deficiency of amplification of the 124‐bp DNA fragment for the WT allele, and^[^ ^3^ ^]^ amplification of the 370‐bp DNA fragment of the Cre gene (Figure S2B, Supporting Information).

### PT‐PRDM16‐KI Mice

The CAG‐LOXP‐EGFP‐stop‐Loxp‐HA‐FCDNA‐GSG‐tdTomato sequence was inserted into the ROSA gene site to produce Rosa26^LSL/+^ mice via homologous recombination using CRISPR/CAS9 technology (obtained from Shanghai Model Organisms) (Figure S4A). Rosa26^LSL/+^XY mice were further crossed with Rosa26^LSL/+^XX mice to produce Rosa 26^LSL/LSL^XY mice, which were crossed with female PEPCK‐Cre (provided by Volker Haase of Vanderbilt University School of Medicine, Nashville, TN) transgenic mice (Rosa26^+/+^X^cre^X^cre^) to produce heterozygous female offspring (Rosa26^LSL/+^X^cre^X), followed by crossing with Rosa26^LSL/+^X^cre^Y males to produce littermate proximal tubule PRDM16 wild‐type (PT‐PRDM16‐WT) and Rosa26^LSL/LSL^X^cre^Y (PT‐PRDM16‐KI) mice (Figure S4B, Supporting Information). Each mouse was subjected to three sets of PCR test. The genotype of PT‐PRDM16‐KI mice was characterized by^[^ ^1^ ^]^ amplification of the 389‐bp DNA fragment for the floxed allele,^[^ ^2^ ^]^ deficiency of amplification of the 994‐bp DNA fragment for the WT allele, and^[^ ^3^ ^]^ amplification of the 370‐bp DNA fragment of the Cre gene (Figure S4C, Supporting Information).

### Mouse Models of Diabetes

For the STZ‐induced model of diabetes, male mice (8–10 weeks of age) were sorted according to their body weight and randomly assigned to control group and treatment group. The treatment group were injected STZ dissolved in 50 mmol L^−1^ sodium citrate buffer (pH 4.5) (50 mg kg^−1^ body weight intraperitoneally daily for 5 consecutive days), and the control group were injected sodium citrate (SC).^[^ ^41^ ^]^ Two consecutive blood glucose readings of more than 200 mg dL^−1^ were considered as diabetic.^[^ ^42^ ^]^ After 4 weeks, the body weights and blood glucose levels of mice were measured. The levels of urine albumin, creatinine, and ACR was carried out according to the previous description.^[^ ^43^ ^]^ Kidney capsules were removed and kidneys were further separated into part of cortex and out of medulla under a microscope according to tissue feature.^[^ ^44^ ^]^ A portion of fixed kidney tissues was used for histopathological analysis by HE staining, Masson staining, and immunohistochemistry. Another portion was used for western blot. Eight‐week‐old db/m and db/db diabetic mice were injected with adeno‐associated virus 2 (AAV2) carrying TRPA1 knockdown and overexpression plasmids and ADV carrying PRDM16 overexpression plasmids through vein tail once for four weeks, AAV2 or ADV carrying negative control sequence (NC) or vector plasmid used as a control.^[^ ^45^ ^]^ Eight‐week‐old db/db diabetic mice were intragastrically administered formononetin at a dose of 25 mg kg^−1^ once a day for four weeks. The animals were euthanized two weeks later and their kidneys were harvested for examination. Each experimental group included six mice.

### Cell Culture and Treatments

The Boston University mouse proximal tubular cell line (BUMPT) was originally provided by Drs. Wilfred Lieberthal and John Schwartz at Boston University School of Medicine (Boston, MA).^[^ ^46^ ^]^ Retrovirus PRDM16 (plasmid 15 504) and PRDM16 shRNA (plasmid 15 505) constructs were purchased from Addgene according to previously described.^[^ ^47^ ^]^ The PRDM16‐RFP stably expressed cell line was generated. Briefly, BUMPT cells were seeded in six‐well plates with DMEM containing 10% FBS and 1% penicillin‒streptomycin (Gibco, 15 140 163) at 37°C in 5% CO2. PB‐TRE‐PRDM16‐RFP (2 ug), PL623 (1 ug), and rtTA (1 ug) were transfected using Lipofectamine 2000 after plating for 24 h (Invitrogen) according to the manufacturer's instructions. After transfection for 24 h, 2 µg mL^−1^ puromycin was then added for 1 week, followed by selection of monoclonal cells that stably expressed PRDM16‐RFP.For experiment, BUMPT cells or the cell line stably expressing PRDM16‐RFP were treated by NG (5 mm D‐glucose), HG (30 mm D‐glucose), or mannitol (5 mm glucose+25 mm D‐mannitol) treatment for 24–48 h. BUMPT cells were transfected with AAV2 carrying TRPA1 knockdown and overexpression plasmids,negative control sequence (NC) and AAV2 vector used as a control, and then treated with NG or HG for 48 h.

### Human Samples

The study was approved by the Review Board of the Second Xiangya Hospital, People's Republic of China (No. Z0037). 19 patients were recruited for this study. All participants were recruited with written informed consent prior to inclusion in the study. Human kidney samples were collected from 9 patients with DKD (including 3 with early stage DKD and 3 late stage DKD), or 10 patients with kidney cancers (PC, *n* = 10) at the Second Xiangya Hospital, Changsha, China(Table S1, Supporting Information).

For DKD samples, the inclusion criteria were^[^ ^1^ ^]^ pathologically diagnosed as a patient with clear diabetic kidney diseases;^[^ ^2^ ^]^ the patient does not have tumor and other kidney diseases;^[^ ^3^ ^]^ serum creatinine was abnormal;^[^ ^4^ ^]^ UAE > 200 µg min^−1^;^[^ ^5^ ^]^ age < 75 years old, and the exclusion criteria were^[^ ^1^ ^]^ patients with tumors found in postoperative medical examinations;^[^ ^2^ ^]^ patients whose blood creatinine was normal. Early stage DKD and late stage DKD were indicated by renal function (GFR) decline and the severity of proteinuria (Table S4, Supporting Information).

Part of the kidney samples were fixed with 4% buffered paraformaldehyde, and then treated with staining of HE, Masson's trichrome, and immunohistochemistry according to previous work.^[^ ^45^, ^48^, ^49^ ^]^ The remainders of the specimens were used to extract proteins for immunoblot analysis.

### Antibodies and Reagents

PRDM16 (PA5‐20872) antibody was obtained from Invitrogen (Carlsbad, CA, USA). TRPA1 (DF13269) antibody and Collagen І (AF7001) antibody(for immunoblotting) were purchased from Affinity (Las Vegas, USA). P38 (8690) antibody, phospho‐P38 (4511) antibody, phospho‐ERK1/2(4370) antibody, ERK1/2 (4695) antibody , HA‐Tag (3724) antibody, phospho‐p65 (3033) antibody, and p65 (8242) antibody were obtained from Cell Signaling Technology (Danvers, MA, USA). β‐tubulin(10094‐1‐AP)antibody, fibronectin(15613‐1‐AP) antibody, and α‐SMA(14395‐1‐AP) antibody were purchased from Proteintech (Rosemont, IL, USA). Collagen І (ab88147) antibody (for immunohistochemistry), Collagen IV(ab6586) antibody, and anti‐TGF‐β1(ab215715) antibody were obtained from Abcam (Cambridge Science Park, Cambridge, UK). All secondary antibodies were obtained from Thermo Fisher Scientific (Waltham, MA, USA). The plasmids of PRDM16‐full length and TRPA1 were generated by the Shenggong Biology Company (Shanghai, China). siRNAs against PRDM16 and TRPA1 were synthesized by the Ruibo Biology Company (Guangdong, Guangzhou, China). Streptozotocin (STZ) were obtained from Sigma Chemical Co. (St.Louis, MO, USA). P38MAPK inhibitor (SB203580), ERK1/2 inhibitor (PD98059), NF‐κ B inhibitor (TPCA‐1), P38MAPK activator (Dehydrocorydaline chloride) and ERK1/2 activator (Honokiol) were purchased from MedChemExpress USA (Deer Park, NJ, USA). AAV2‐mediated TRPA1 knockdown and overexpression systems and ADV‐PRDM16 overexpression were purchased from the Heyuan Biology Company (Shanghai, China). TGF‐β1 ELISA Kit was purchased from Proteintech (Rosemont, IL, USA). Formononetin was obtained from Guangzhou Institutes of Biomedicine and Health.

### ChIP‐Seq

The BUMPT cell line stably expressing HA‐PRDM16‐RFP was treated with doxycycline (DOX) with NG or HG with for 48 h. Then these samples were harvested for ChIP‐sequencing. Anti‐HA‐tag antibodies were used to precipitate protein‐bound DNA. DNA samples were end‐repaired, A tailed, and adaptor ligated using TruSeq Nano DNA Sample Prep Kit (#FC‐121‐4002, Illumina), following the manufacturer's instructions.^[^ ^49^ ^]^ Approximately 200–1500 bp fragments were size selected using AMPure XP beads. The final size of the library was confirmed by Agilent 2100 Bioanalyzer. The libraries were then sequenced on the Illumina HiSeq 4000 following the HiSeq 3000/4000 SBS Kit (300 cycles) protocol.^[^ ^50^ ^]^

Sequence quality was examined using the FastQC software. ChIP–seq Reads were aligned to Mouse genome (MM10) using BOWTIE software (V2.1.0).^[^ ^51^ ^]^ Peak detection used MACS V1.4.2 (Model‐based Analysis of ChIP‐Seq) software.^[^ ^52^ ^]^ Statistically significant ChIP‐enrichment peaks were identified by comparison of IP versus Input or comparison to a Poisson background model (Cut‐off p‐value = 10^−3^). The annotation of the peaks which were located within −2Kb to +2Kb around the corresponding gene TSS. The gene body includes all exons, introns, promoters, upstream, and intergenic^[^ ^49^ ^]^ (Table S2, Supporting Information).

### ChIP Analysis

ChIP assays were carried out using a ChIP kit (Millipore, Boston, MA, USA). Anti‐HA‐tag antibodies and phospho‐p65 were used to precipitate protein‐bound DNA. Mouse IgG was used as a control. Precipitated DNAs were detected by PCR using specific primers:

TRPA1‐1(P1): 5′‐AGGCACGACACAGTCTTGTTCATG‐3′(forward)

And 5′‐CCACACAGGCTATGGGGAAGAAAAG‐3′(reverse);

TRPA1‐2(P2): 5′‐TGTAGCTCTGAGTTCTTTCCCAGT‐3′(forward)

And 5′‐ACCCACCAGAAGAAGAGGCT‐3′(reverse);

TRPA1‐3(P3): 5′‐AGATTCTTCCGTGCCATTGCCTTC‐3′(forward)

and 5′‐CAGAGGTGGAAATGCCAGAGAGC–3′(reverse);

TRPA1‐4(P4): 5′‐TGTAGGGCGTCTGACAGTCCATC‐3′(forward)

and 5′‐TGAATGTATGTGGCGGCAAGAA GG −3′ (reverse).

PRDM16‐1(P1): 5′‐ GGAGAGAGCGTCGGCTAGTTTC‐3′(forward)

and 5′‐ AGGAGCTGGGGTCCTTTCGG‐3′ (reverse).

PRDM16‐1(P2): 5′‐ AGGAAGATCAGGGTCCCTCGTTAG‐3′(forward)

and 5′‐ GCAGAGAAGGCTCAAGGTCACC −3′ (reverse).

PRDM16‐1(P3): 5′‐ GGACCTTGGATTTCGGAGAGAGTG‐3′(forward)

and 5′‐ CGAGTACGGCTACGATTTGAGTTTG −3′ (reverse).

### Luciferase Reporter Assay

The five segments of TRPA1 promoter luciferase reporter plasmids were constructed by Shenggong Biology Company (Shanghai, China). P4‐1: −500‐0,P4‐2: −400‐0, P4‐3:−300‐0, P4‐4:−200‐0, P4‐5:−100‐0). The promoter sequences used in the luciferase reporter assays were provided in Supplementary Table S3. These TRPA1 promoter plasmids were separately transfected into the stable HA‐PRDM16‐RFP‐expressing cell line with or without DOX for 48 h. The PGMLR‐TK luciferase reporter was used as a control vector. Luciferase activities were then measured using a SpectraMax M5 (Molecular Devices, Sunnyvale, CA, USA) and normalized according to pGMLR‐TK activity.

### Reverse Transcription Quantitative PCR (RT‐qPCR)

Total RNA was extracted from BUMPT cells or kidney tissue using the Trizol reagent (Invitrogen, Carlsbad, CA, USA), and then reverse‐transcripted into first‐strand cDNA using the Prime Script RT Reagent kit and gDNA Eraser (TaKaRa, RR037A) according to previously described.^[^ ^53^, ^54^ ^]^ Next, the cDNA was used as a template with TB green(TaKaRa, RR820A)and a Light cycler 96 (Roche) with the following primer pairs: PRDM16: 5´‐ CAGCAACCTCCAGCGTCACATC −3´(forward) and 5´‐GCGAAGGTCTTGCCACAGTCAG‐3´(reverse); TRPA1:5´‐ CGTGTGAAGT GCTGAATATAATGGATGG‐3´(forward) and 5´‐TGTTTCTATTTCGGAGGTTTGGATTTGC‐3´ (reverse). β‐actin: 5´‐GTGCTATGTTGCTCTAGACTTCG‐3´(forward) and 5´‐ATGCCACAG GATTCCATACC‐3´(reverse). ΔΔCT values were used to perform the relative quantification. The absolute quantification was carried out according to a standard curve.

### Immunoblot Analysis

For immunoblotting, briefly, equal amounts of proteins were loaded on each lane and separated by SDS‐PAGE, and then transferred onto a nitrocellulose membrane (Amersham, Buckinghamshire, UK).^[^ ^53^ ^]^ The blot was then washed in TBST buffer, blocked for 60 minutes in the blocking buffer (5% milk). The blot was then incubated with primary antibodies against PRDM16(1:1000 dilution), Collagen I(1:1000 dilution) and Collagen IV(1:2000 dilution), fibronectin(1:2000 dilution), a‐SMA(1:2000 dilution), HA(1:2000 dilution), TGF‐β1(1:2000 dilution),TRPA1(1:1000 dilution), phospho‐P38MAPK(1:1000 dilution), P38MAPK(1:1000 dilution), phospho‐ERK1/2(1:1000 dilution), ERK1/2 (1:1000 dilution), phospho‐p65(1:1000 dilution), p65 (1:1000 dilution)and β‐Tubulin(1:2000 dilution) overnight incubation at 4°C. followed by incubation with secondary antibody (1:5000 dilution) and detection reagents. Anti‐β‐tubulin was used as an internal control.

### H&E Staining, Masson's Trichrome Staining, Immunohistochemical Analysis, and Immunofluorescence

H&E staining was used to analyze the histological injury. The glomerular and tubular damage score of kidneys was performed as previously described.^[^ ^42^, ^55^ ^]^ Fibrosis was evaluated by the Masson's trichrome staining. Immunohistochemical analysis was carried out using anti‐collagen I (1:100 dilution), anti‐collagen IV (1:100 dilution), anti‐FN (1:50 dilution), anti‐PRDM16 (1:100), anti‐a‐SMA (1:100 dilution), and phospho‐p65 (1:100 dilution) according to the previous protocol.^[^ ^40^, ^43^, ^45^ ^]^ Stained samples were analyzed using an Olympus microscope equipped with UV epi‐illumination. For immunofluorescence staining, the sections were incubated with specific primary antibody (PRDM16 1:500) overnight at 4°C followed by a secondary fluorescent antibody for 1 hour at 37°C in the dark. DAPI was then added for 3–5 min and the sections observed by fluorescent microscopy.

### Molecular Docking Analysis about the Binding of Formononetin to PRDM16

First, the 3D protein structure of PRDM16 was predicted by AlphaFold^[^ ^56^ ^]^ based on the amino acid sequence of mouse PRDM16. Second, CavityPlus was applied^[^ ^57^ ^]^ to detect all potential drug‐binding pockets on the surface of PRDM16. Third, AutoDock Vina 1.2^[^ ^58^ ^]^ was used to dock formononetin to all potential drug‐binding pockets of PRDM16 and the best drug‐binding pocket was identified based on the docking score function with default parameters.

### Statistics

All data were presented as means ± SD. Two groups were compared using two‐tailed t‐tests. One‐way ANOVA was used for multiple group comparisons. Kruskal‐Wallis test was used for the non‐parametric tests for multiple group's comparison. For all statistical tests, *P* values <0.05 were considered significant.

## Conflict of Interest

The authors declare no conflict of interest.

## Author Contributions

D.S.Z. and Z.D. conceived and designed the experiments. F.X. carried out the experiments. F.X., H.W.J., X.Z.L., J.P., H.L.L., L.X. W., P.Z., and J.X.C. analyzed the data. S.F.Q. contributed reagents and materials. Y.X.X. and Y.J.L. contributed the analysis tools. D.S.Z. and Z.D. wrote the main manuscript but all authors reviewed the manuscript.

## Supporting information

Supporting Information

## Data Availability

The data that support the findings of this study are available in the supplementary material of this article.
