# Supplementary material for: Discovery of PRDM16‐Mediated TRPA1 Induction as the Mechanism for Low Tubulo‐Interstitial Fibrosis in Diabetic Kidney Disease
Source: Adv Sci (Weinh). 2023 Dec 10;11(7):2306704. doi: 10.1002/advs.202306704 (PMC10870028; doi:10.1002/advs.202306704)
Supplement: Supplementary file 1 — Supporting Information [file ADVS-11-2306704-s001.pdf]

ADVANCED SCIENCE
Open Access

Supporting Information

for *Adv. Sci.*, DOI 10.1002/advs.202306704

Discovery of PRDM16-Mediated TRPA1 Induction as the Mechanism for Low Tubulo-Interstitial Fibrosis in Diabetic Kidney Disease

Fang Xu, Hongwei Jiang, Xiaozhou Li, Jian Pan, Huiling Li, Luxiang Wang, Pan Zhang, Junxiang Chen, Shuangfa Qiu, Yuxin Xie, Yijian Li, Dongshan Zhang and Zheng Dong**

Supplementary Data

(12figures, 4 tables)

Discovery of PRDM16-mediated TRPA1 induction as the mechanism for low tubulo-interstitial fibrosis in diabetic kidney disease

Fang Xu[1,2,5], Hongwei Jiang[7],Xiaozhou Li[1,2], Jian Pan[1,2], Huiling Li[4],Luxiang Wang[1,2], Pan Zhang[1,2,6], Junxiang Chen[5],Shuangfa Qiu[1,2],Yuxin Xie[1,2], Yijian Li[3], Dongshan Zhang [1,2,5,7*], Zheng Dong[5,8*]

[1] Department of Emergency Medicine, [2] Emergency Medicine and Difficult Diseases Institute, [3] Department of Urology, [4]Department of Ophthalmology, [5]Department of Nephrology, Second Xiangya Hospital, Central South University, Changsha, Hunan, People's Republic of China. [6]Department of Epidemiology and Health Statistics, Xiangya School of Public Health, Central South University.[7]Department of Endocrinology, First Affiliated Hospital of Henan University of Science and Technology, People's Republic of China.[8]Department of Cellular Biology and Anatomy, Medical College of Georgia at Augusta University, Augusta, Georgia, USA.

Corresponding authors:

Dongshan Zhang: Department of Emergency Medicine, Emergency Medicine and Difficult Diseases Institute, Department of Nephrology, Second Xiangya Hospital, Central South University, Changsha, Hunan 410011, People's Republic of China.

Tel +86 138 7589 9625. Email: dongshanzhang@csu.edu.cn

Zheng Dong: Department of Nephrology, Second Xiangya Hospital, Central South University, Changsha, Hunan 410011, People's Republic of China; Department of Cellular Biology and Anatomy, Medical College of Georgia at Augusta University, Augusta, Georgia, USA.

Tel: 706-7212825; Email: zdong@augusta.edu.

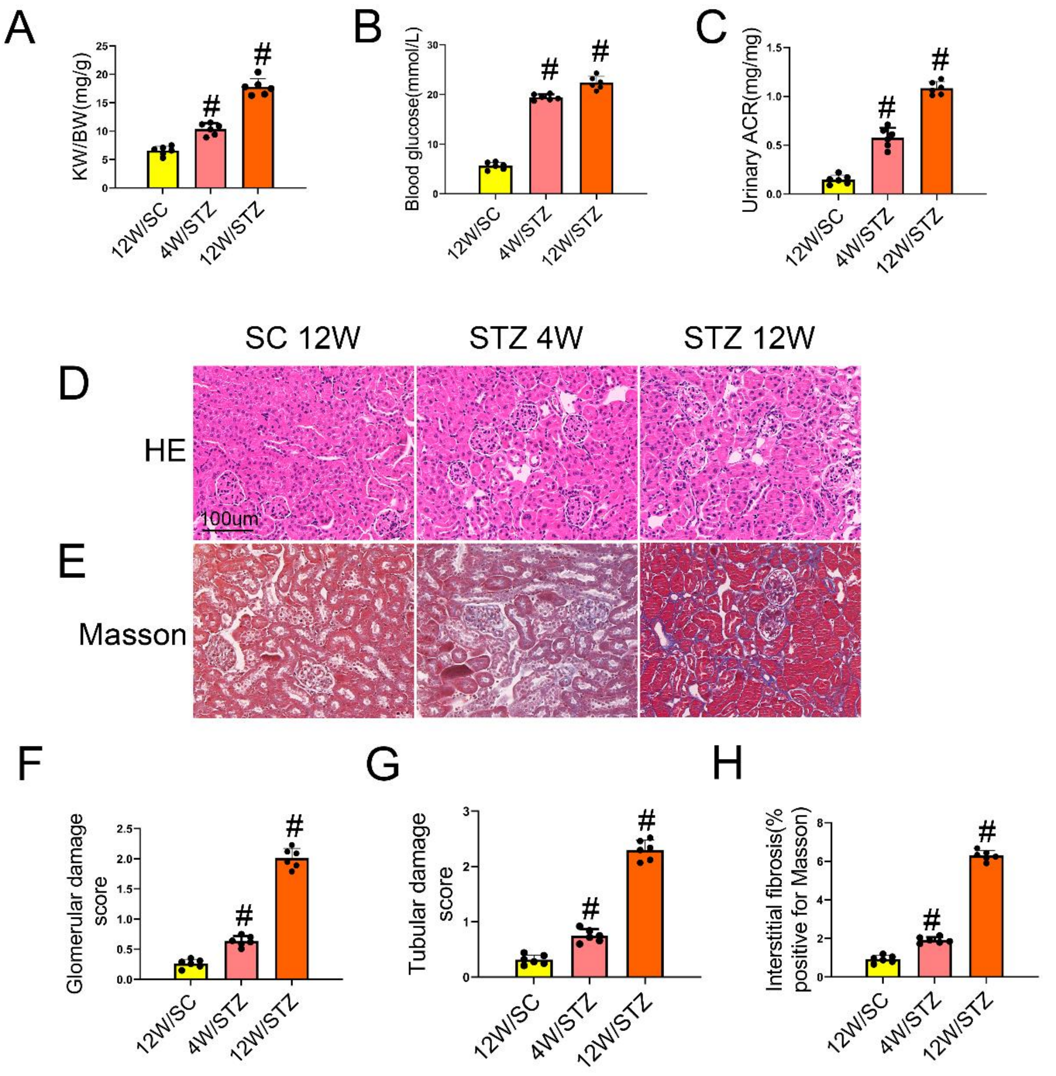

Supplementary Figure 1: STZ-induced diabetes in mice

C57BL/6 mice were intraperitoneally injected with 50 mg/kg body weight STZ for 5 consecutive days to induce diabetes or injected with sodium citrate (SC) as a control. Samples were collected at 12 weeks or 4 weeks after diabetes induction for analysis. (A) kidney to total body weight ratio (KW/BW). (B) Fasting blood glucose. (C) Urinary albumin to creatinine ratio (ACR). (D) HE staining of renal tissues. (E)Masson staining of renal tissues. (F) Quantification of glomerular damage. (G) Quantification of tubular damage. (H) Quantification of tubulointerstitial fibrosis in the kidney cortex. Original magnification x 400. Scar Bar:100μM. Data are expressed as mean ± SD (n = 6). A-C&F-H: #P < 0.05,12 weeks SC groups versus STZ 4W groups or STZ 12W groups.

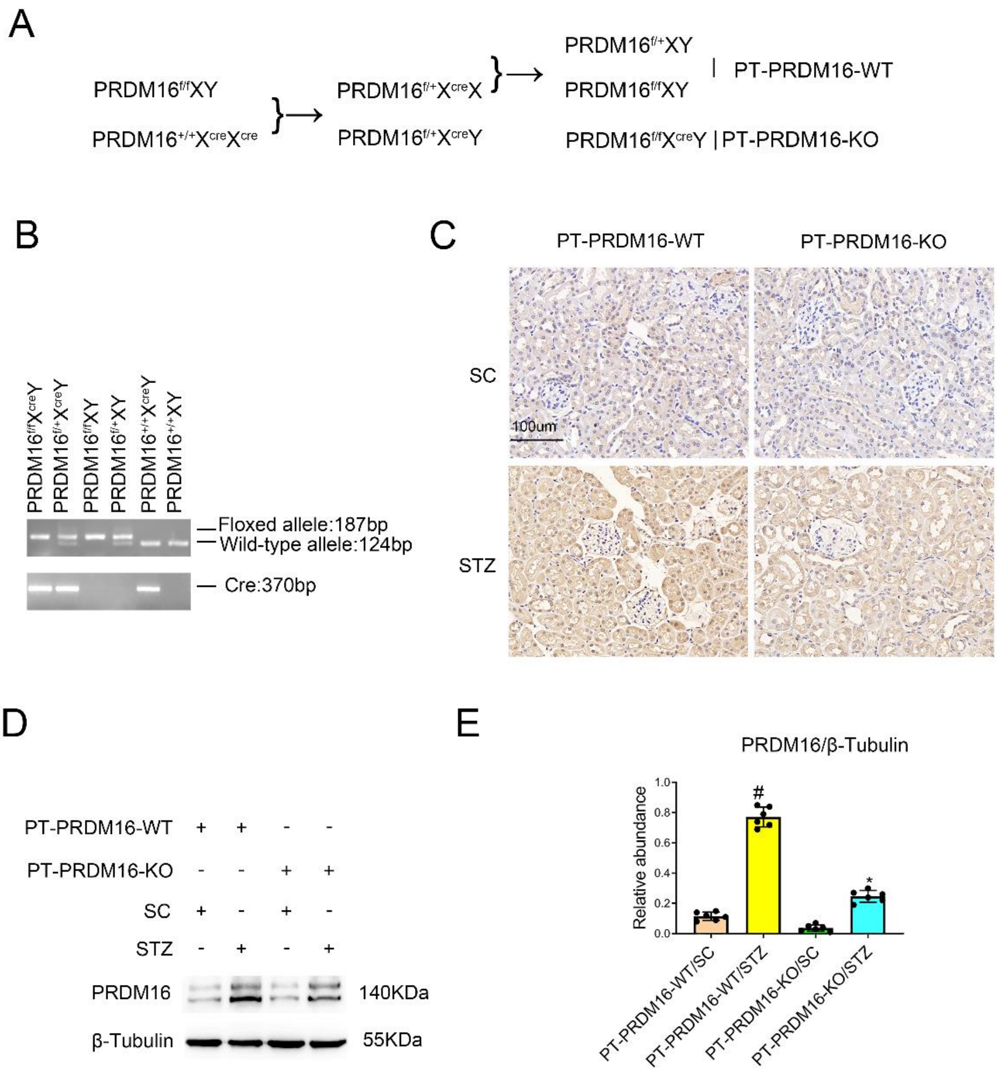

Supplementary Figure 2: Creation and characterization of PT-PRDM16-KO mouse model. (A) Breeding protocol for the creation of PT-PRDM16-KO mice. (B) PCR detection of wild-type and floxed alleles of PRDM16 and PEPCK-Cre allele. Littermate mice of PT-PRDM16-WT and PT-PRDM16-KO were intraperitoneally injected with 50 mg/kg body weight STZ for 5 consecutive days or sodium citrate (SC) as a control. The fasting blood glucose levels of more than 200 mg/dL for two consecutive measurements were regarded as diabetic. Samples were collected 12 weeks after diabetes induction for analysis. (C) Immunohistochemical staining of PRDM16. (D) Immunoblot analysis of PRDM16 and β-tubulin. (E) Densitometry analysis of immunoblot bands. Data are expressed as mean ±SD (n = 6). Original magnification x 400. Scale Bar:100μM. # *P<0.05*, versus SC-treated PT-PRDM16-WT group; * *P<0.05*, versus STZ-treated PT-PRDM16-WT group.

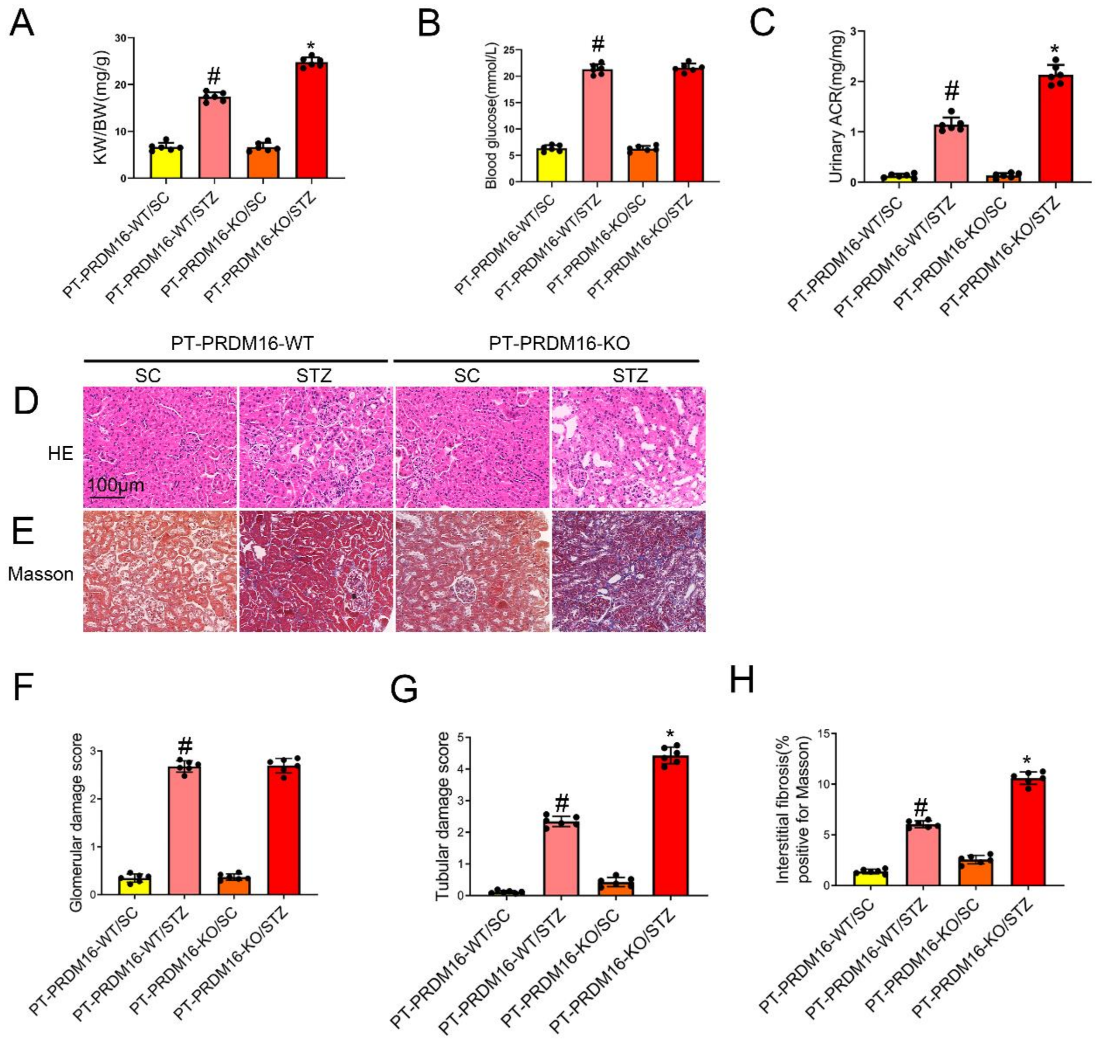

Supplementary Figure 3: PRDM16-knockout from kidney proximal tubules enhances renal fibrosis in STZ-induced diabetic mice.

Littermate mice of PT-PRDM16-WT and PT-PRDM16-KO were intraperitoneally injected with 50 mg/kg body weight STZ for 5 consecutive days or sodium citrate (SC) as a control. The fasting blood glucose levels of more than 200 mg/dL for two consecutive measurements were regarded as diabetic. Samples were collected 12 weeks after diabetes induction for analysis. (A) kidney to total body weight ratio (KW/BW). (B) Fasting blood glucose. (C) Urinary albumin to creatinine ratio (ACR). (D) HE staining of renal tissues. (E)Masson staining of renal tissues. (F) Quantification of glomerular damage. (G) Quantification of tubular damage. (H) Quantification of tubulointerstitial fibrosis in the kidney cortex. Original magnification x 400. Scale Bar:100μM. Data are expressed as mean ± SD (n = 6). # *P<0.05*, versus SC-treated PT-PRDM16-WT group * *P<0.05*, versus STZ-treated PT-PRDM16-WT group.

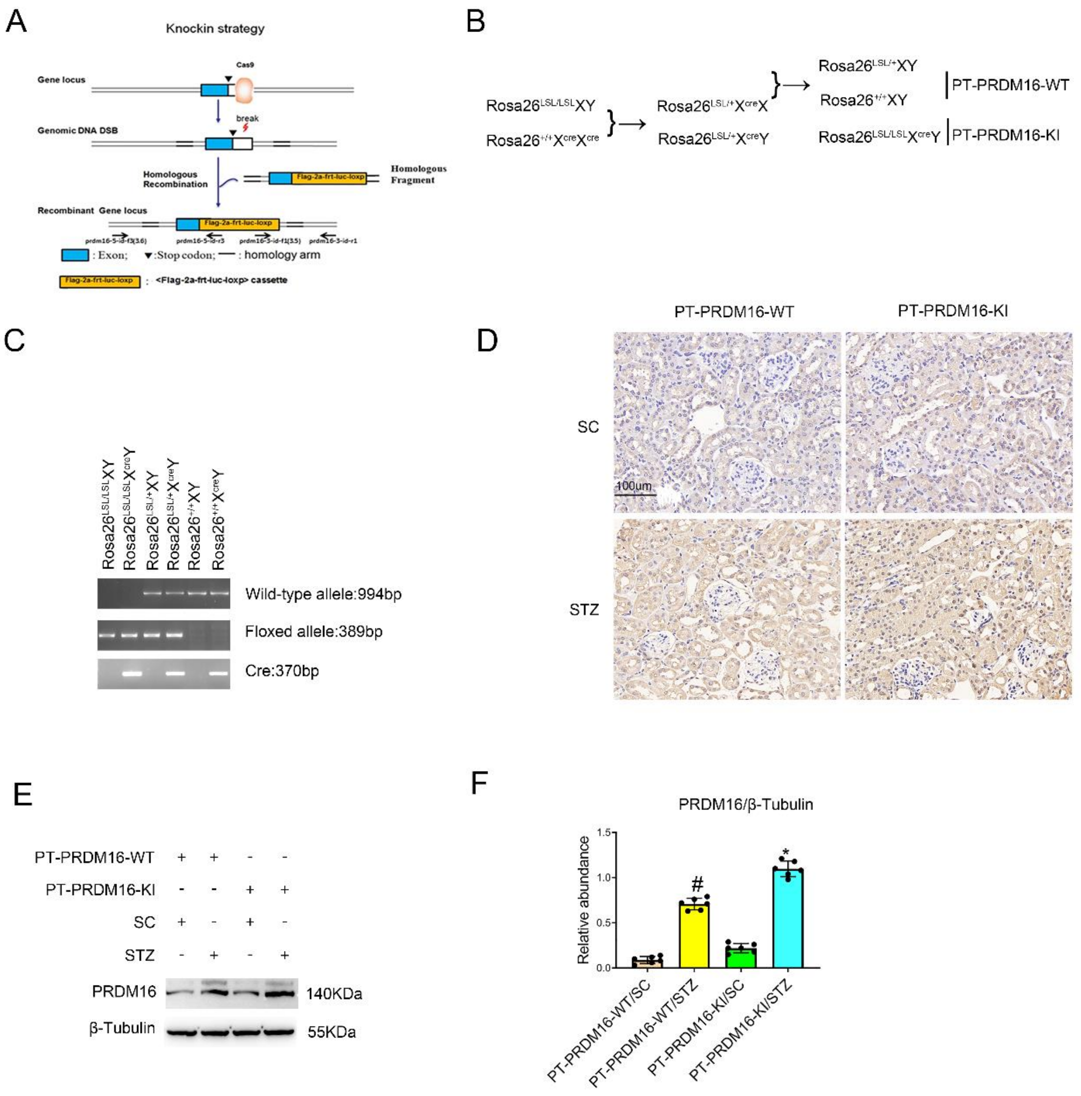

Supplementary Figure 4: Creation and characterization of the PT-PRDM16-KI mouse model. (A) The strategy of PT-PRDM16 knock-in. (B) Breeding protocol for the creation of PT-PRDM16-KI mice. (C) PCR detection of genotype of wild-type and floxed alleles of PRDM16 and PEPCK-Cre allele. The mice were intraperitoneally injected with 50 mg/kg body weight STZ for 5 consecutive days or sodium citrate (SC) as a control. Samples were collected 12 weeks after diabetes induction for analysis. (D) Immunohistochemical staining of PRDM16. (E) Immunoblot analysis of PRDM16 and β-tubulin. (F) Densitometry analysis of immunoblot bands. Data are expressed as mean ± SD (n = 6). Original magnification x 400. Scale Bar:100μM. # *P<0.05*, versus SC-treated PT-PRDM16-WT group * *P<0.05*, versus STZ-treated PT-PRDM16-WT group.

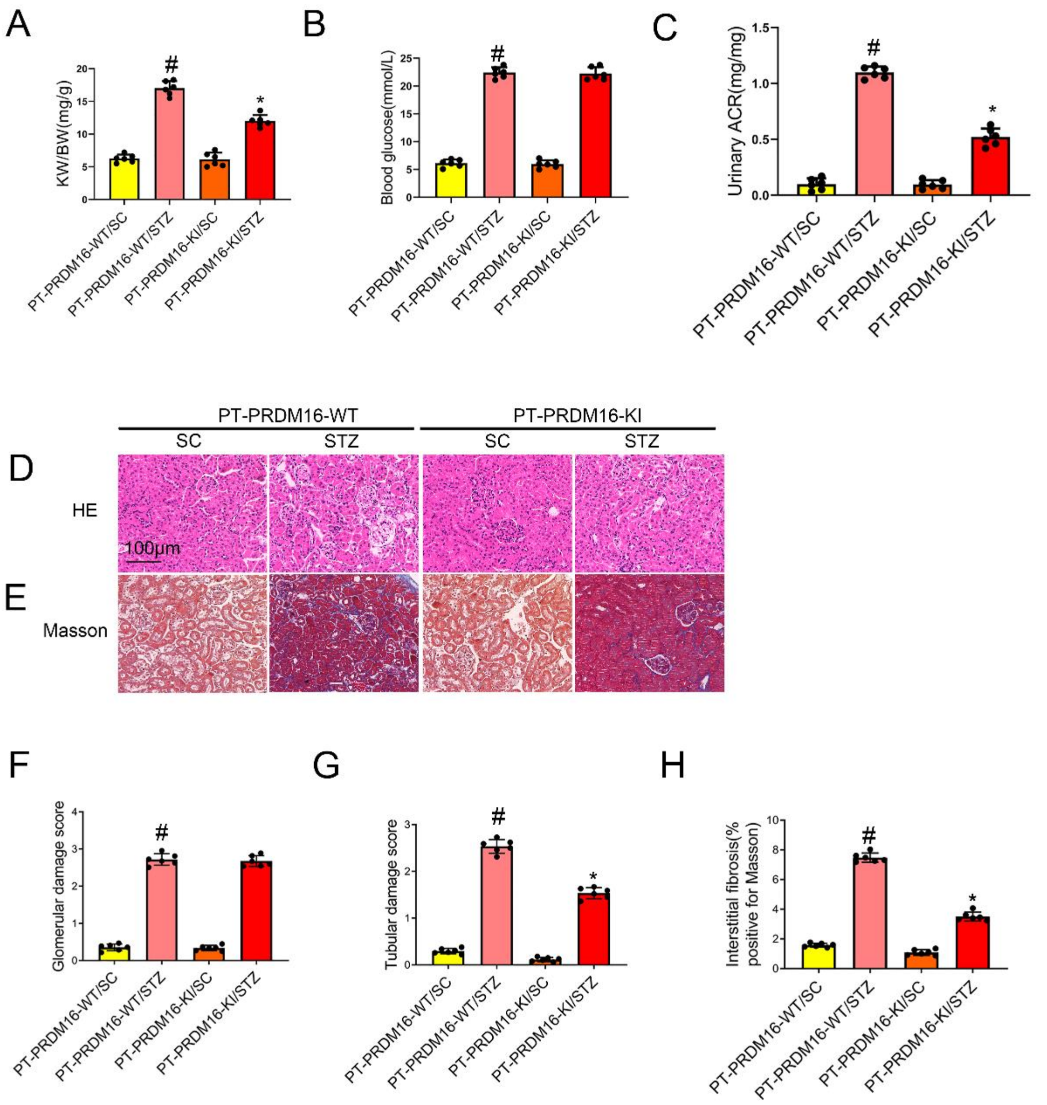

Supplementary Figure 5: PRDM16 overexpression in kidney proximal tubules attenuates renal fibrosis in STZ-induced diabetic mice.

Littermate mice of PT-PRDM16-WT and PT-PRDM16-KI were intraperitoneally injected with 50 mg/kg body weight STZ for 5 consecutive days or sodium citrate (SC) as a control. The fasting blood glucose levels of more than 200 mg/dL for two consecutive measured readings were regarded as diabetic. Samples were collected 12 weeks after diabetes induction for analysis. (**A**) KW/BW. (**B**) Fasting blood glucose. (**C**) Urinary ACR. (**D**) Representative images of HE staining. (**E**) Representative images of Masson staining. (**F**) Quantification of glomerular damage score. (**G**) Quantification of tubular damage score. (**H**) Quantification of tubulointerstitial fibrosis in the kidney cortex. Original magnification x 400. Scale Bar:100μM. Data are expressed as mean ± SD (n = 6). # *P<0.05,* versus SC-treated PT-PRDM16-WT group * *P<0.05*, versus STZ-treated PT-PRDM16-WT group.

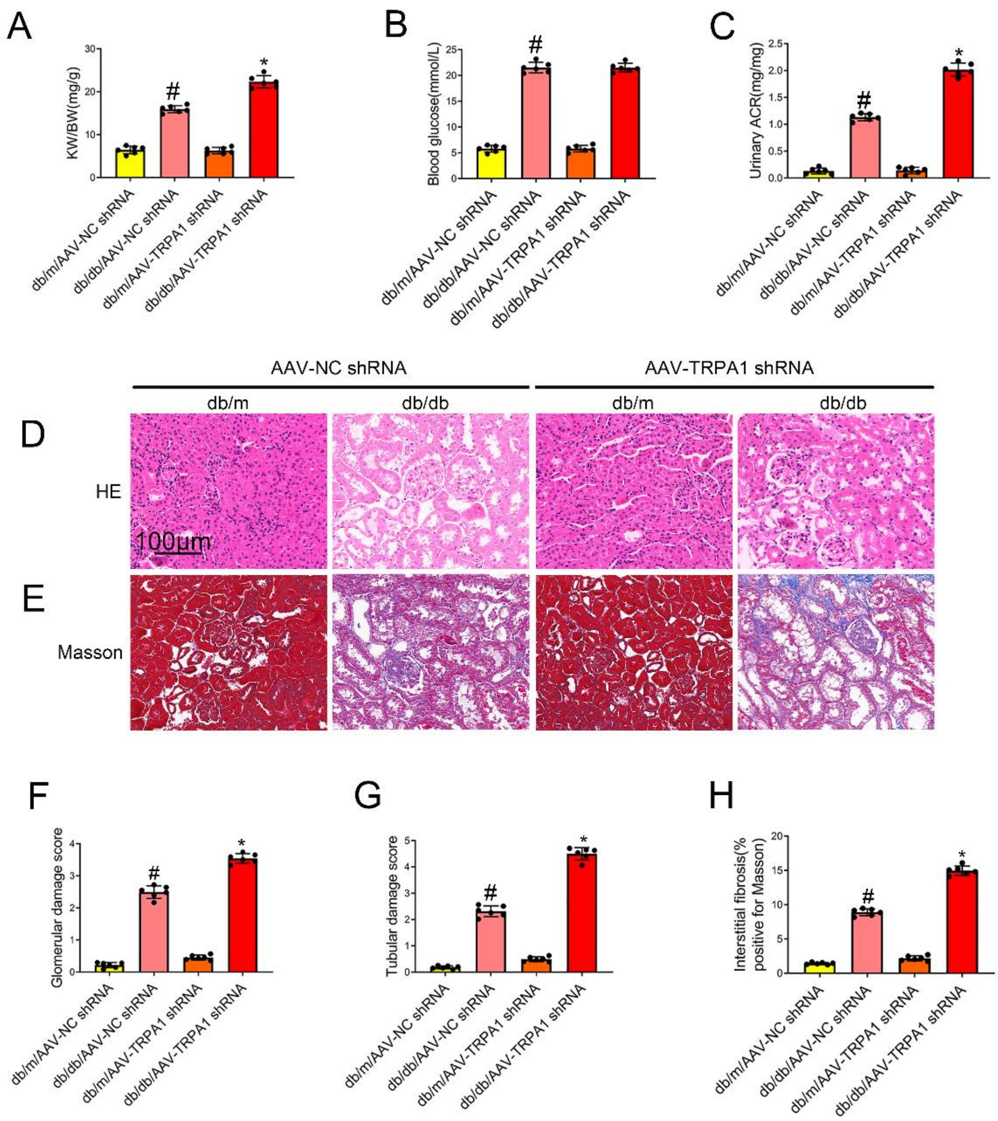

Supplementary Figure 6: Knockdown of TRPA1 enhances renal fibrosis in db/db diabetic mice. Eight-week-old db/db diabetic mice were injected with AAV2 carrying shRNA or negative control sequence (NC) through vein tail for four weeks. (**A**) kidney to body weight ratio (KW/BW). (**B**) Fasting blood glucose. (**C**) Urinary ACR. (**D**) Representative images of H&E staining. (**E**) Representative images of Masson staining. (**F**) Quantification of glomerular damage score. (**G**) Quantification of tubular damage score. (H) Quantification of tubulointerstitial fibrosis in the kidney cortex. Original magnification x 400. Scale Bar:100μM. Data are expressed as mean ± SD (n = 6). # *P<0.05*, versus db/m/NC group. * *P<0.05*, versus db/db/NC group.

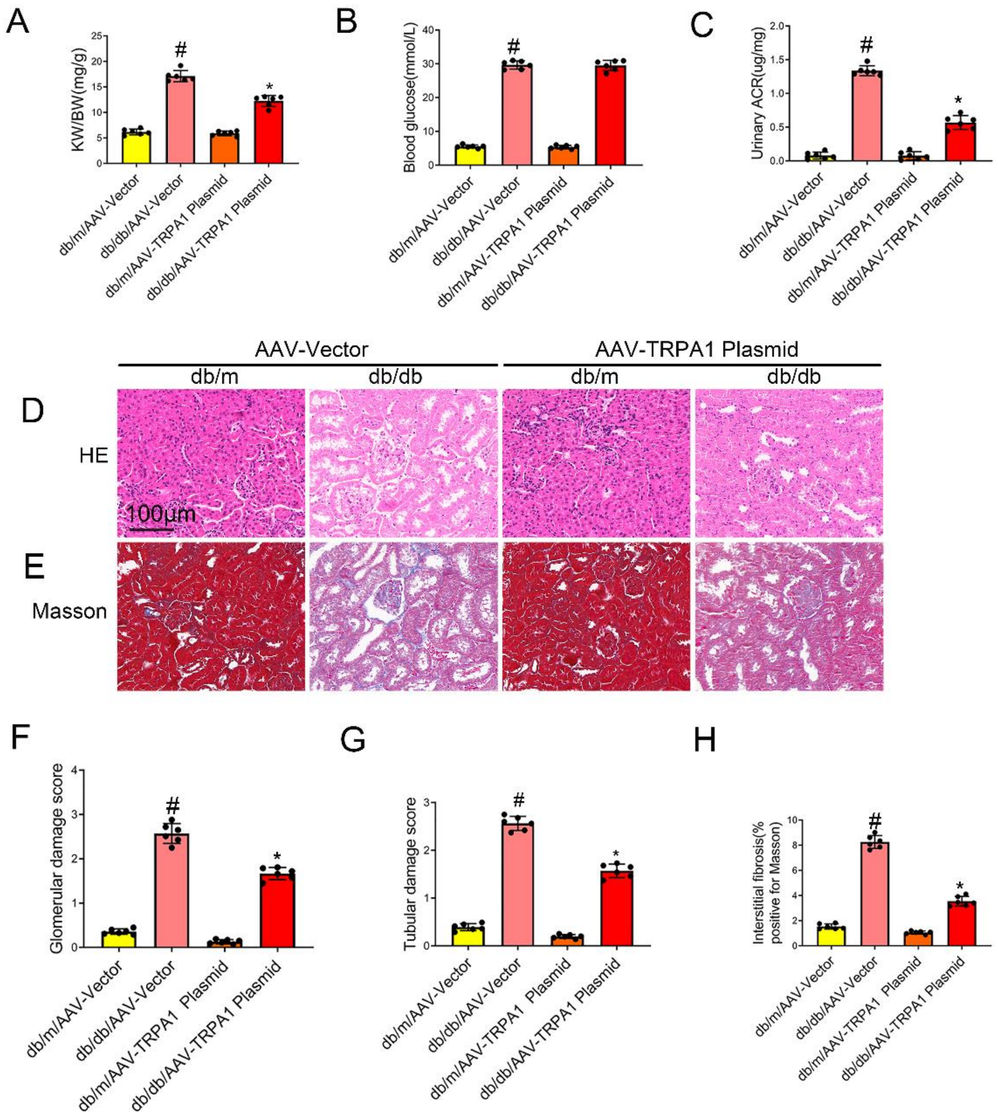

Supplementary Figure 7: AAV2-mediated TRPA1 overexpression attenuates renal fibrosis in db/db diabetic mice.

Eight-week-old db/db diabetic mice were injected with AAV2 carrying TRPA1 overexpression plasmid or AAV2 empty vector through vein tail for four weeks. (**A**) KW/BW. (**B**) Fasting blood glucose. (**C**) Urinary ACR. (**D**) Representative images of H&E staining. (**E**) Representative images of Masson staining. (**F**) Quantification of glomerular damage score. (**G**) Quantification of tubular damage score. (**H**) Quantification of tubulointerstitial fibrosis in the kidney cortex. Original magnification x 400. Scale Bar:100μM. Data are expressed as mean ± SD (n = 6). # *P<0.05*, versus db/m/AAV-Vector group. * *P<0.05*, versus db/db/AAV-Vector group.

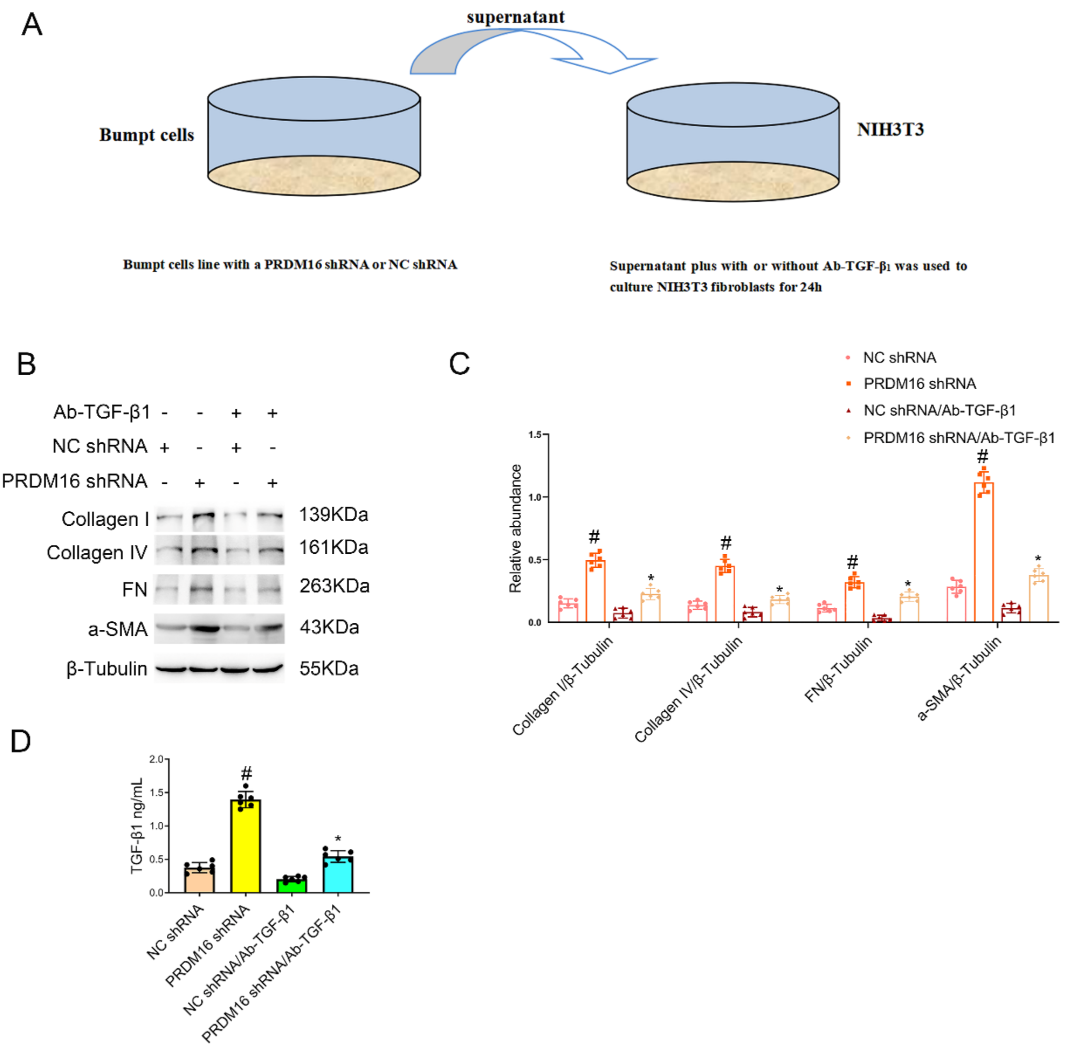

Supplementary Figure 8: Culture medium of BUMPT cells transfected with PRDM16 induces fibrotic protein expression in NIH 3T3 fibroblasts via secretion of TGF-β1.

The NC shRNA or PRDM16 shRNA BUMPT cells were treated with or without Ab-TGF-β1 for 24 h to collect their culture media, which were separately transferred to 3T3 fibroblasts for another 24 h. (A) Diagram of the medium transfer model. (B)Immunoblot analysis of Col 1&IV, FN, α-SMA, and β-tubulin. (C)Analysis of the gray-scale image between them. (D) Concentration of TGF-β1 by ELISA. Data are expressed as means ± SD (n = 6). # *P<0.05* versus the Vector group. **P<0.05* versus PRDM16 group.

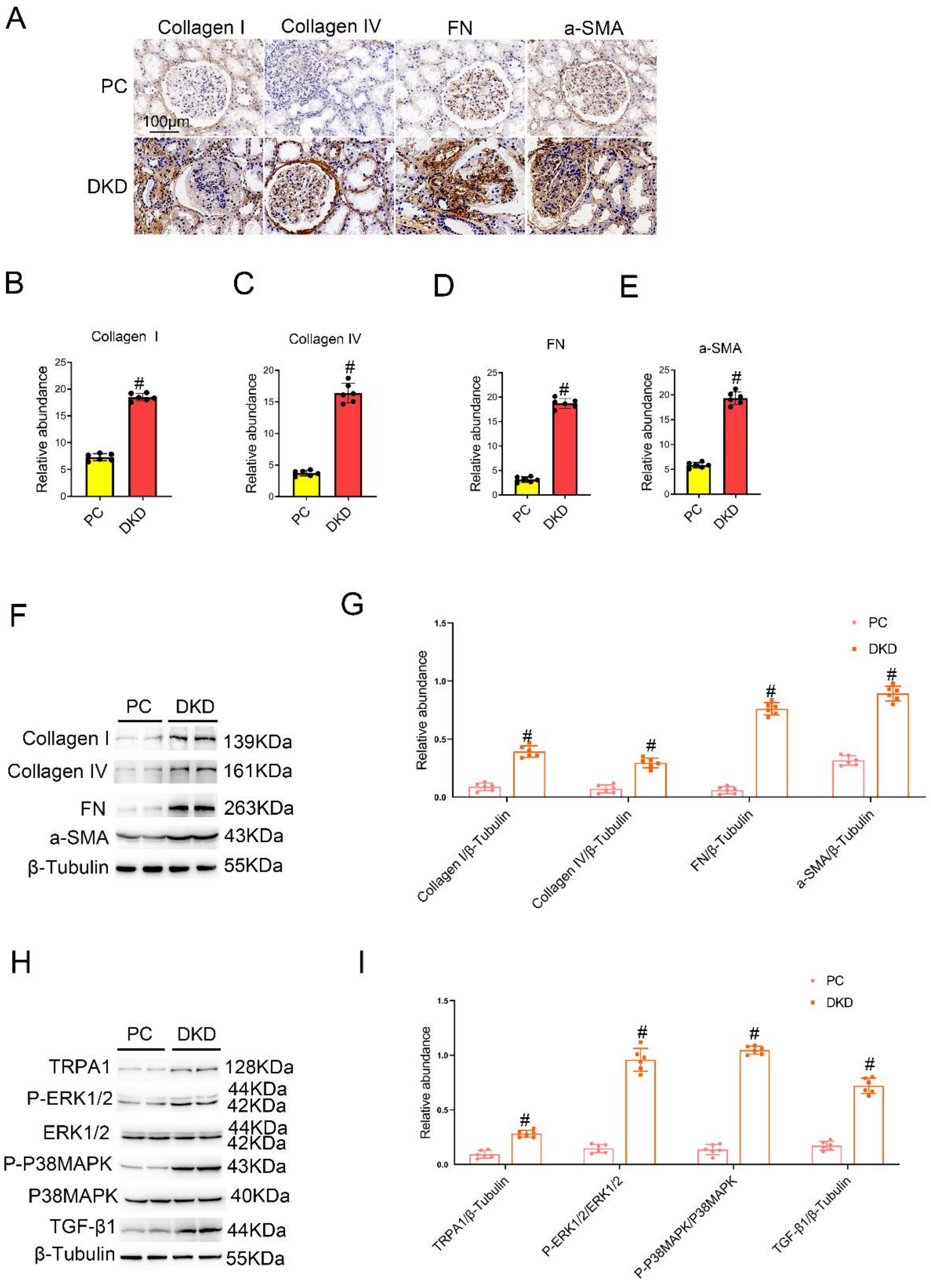

Supplementary Figure 9: PRDM16/TRPA1/MAPK/TGF-β1 axis in kidney biopsies of human DKD patients.

(**A**) Immunohistochemistry staining of fibrotic proteins. (**B-E**) Quantify immunohistochemistry staining. (**F**) Immunoblot analysis of Collagen I&IV, FN, a-SMA, and β-tubulin. (**G**) Densitometry analysis of immunoblot bands. (**H**) Immunoblots analysis of TRPA1, MAPK activation and TGF-β1 expression. (**I**) Densitometry analysis of immunoblot bands. Original magnification x400. Data are expressed as mean ± SD (n = 6). #p < 0.05, DKD groups versus PC groups.

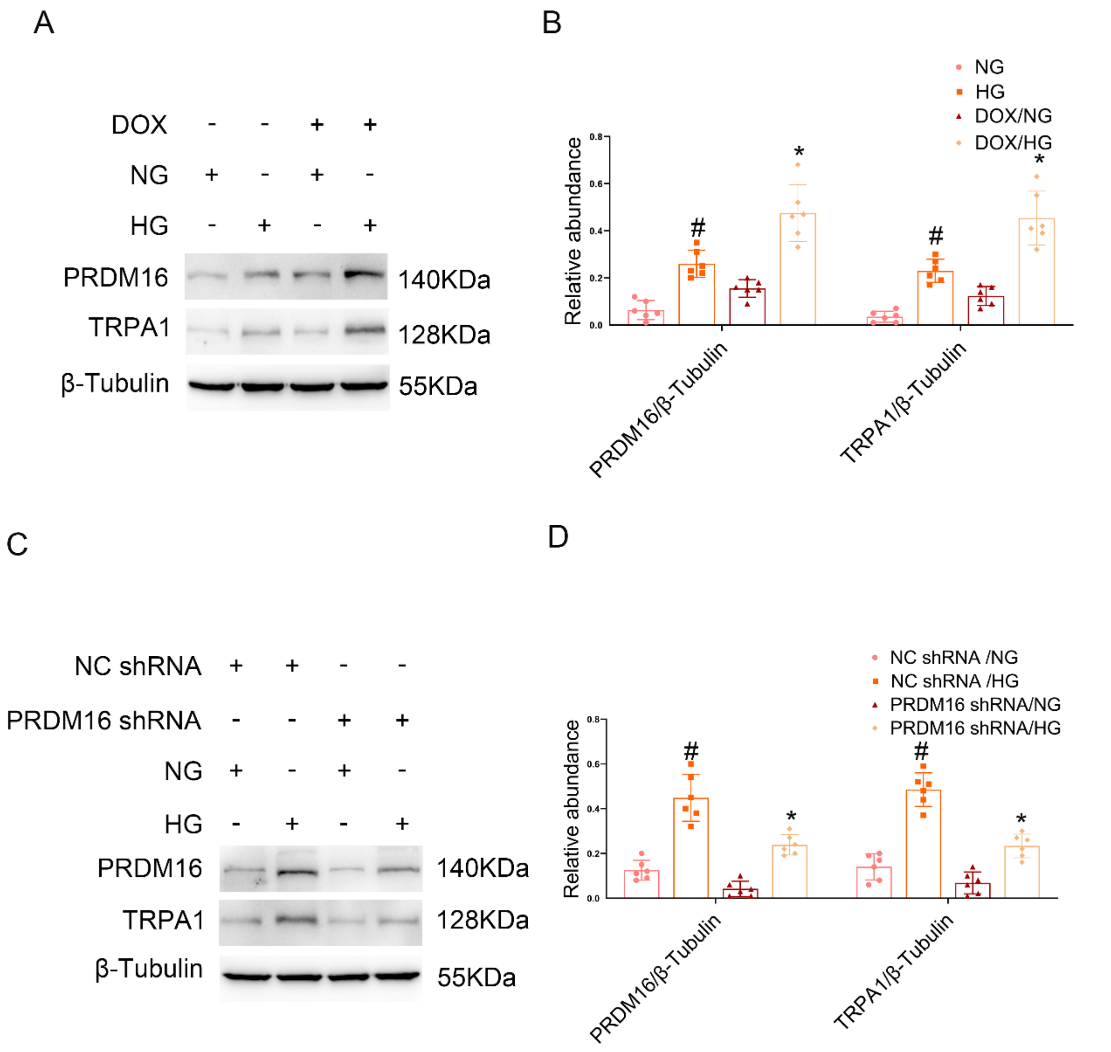

Supplementary Figure 10: PRDM16 suppresses HG-induced and STZ-induced MAPK activation and TGF-β1 expression in renal tubular cells upstream of TRPA1.

(**A, B**) PRDM16-RFP stably transfected cells with or without DOX induction were treated with NG or HG 48 h to collect lysate. (**A**) Immunoblot analysis of PRDM16 and TRPA1. (**B**) Densitometry analysis of immunoblot bands. (**C, D**) PRDM16 was knocked down with shRNAs in BUMPT cells and then subjected the cells to HG or NG incubation. (**C**) Immunoblot analysis of PRDM16 and TRPA1. (**D**) Densitometry analysis of immunoblot bands. Data are expressed as mean ± SD (n = 6). # *P<0.05*, versus scramble with NG group. * *P<0.05*, versus scramble with HG group.

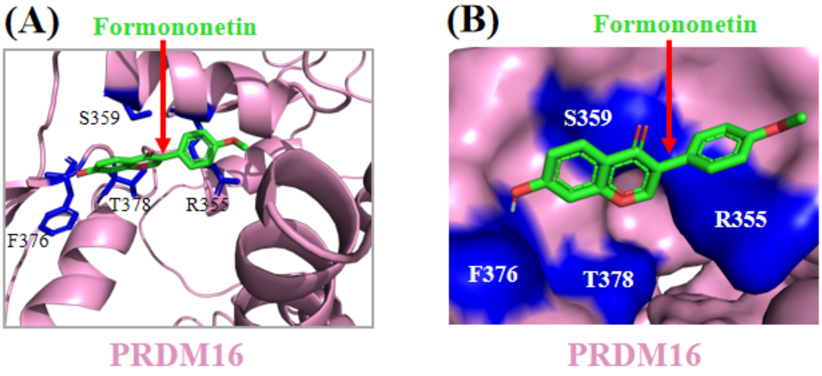

Supplementary Figure 11: The molecular docking analysis to reveal the drug binding of formononetin to PRDM16.

Drug binding pocket of formononetin in PRDM16. The cartoon and surface representations are shown on the left (A) and right (B), respectively.

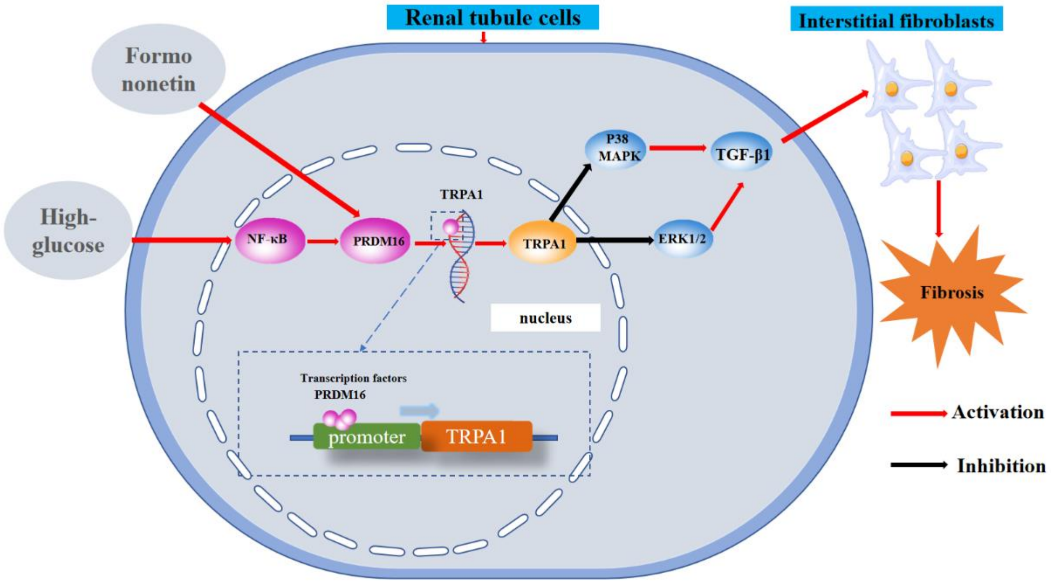

Supplementary Figure 12: The anti-fibrotic pathway of NF-κB/PRDM16/TRPA1/MAPK/TGF-β1 activated in renal tubular cells in diabetes.

High glucose in diabetes induces PRDM16 via NF-κB in kidney tubule cells, which may transcriptionally upregulate TRPA1. TRPA1 then suppresses p38 and ERK1/2 MAPKs to block TGF-β1 expression and secretion, resulting in the inhibition of tubulo-interstitial fibrosis in the early stage of DKD.

Supplementary Table 1. The basic clinical information of PC and DKD patients

Variable	PC (10)	DKD (9)
Age(years) (mean)[SD]	48.8±5.39	55.4±6.35
Gender (Male:Female)	5 ： 5	4 ： 5
Serum Glucose(mmol/L) (mean)[SD]	5±0.28	10.3±0.97#
eGFR ml/min/1.73 m^2(mean) [SD]	101.2±1.40	47.8±22.1#
Urine Protein		+：4
	-	++： 2
		+++： 3

Note:— indicate the lack of proteinuria.

Data are expressed as mean ± SD (n = 6). #p < 0.05, DKD groups versus PC groups.

Supplementary Table 2 Genes with PRDM16 binding to their promoter regions

Genes with increased PRDM16 binding to promoter after high glucose incubation

4930502A04Rik	Il17f	Ankrd49	Olfr19
Mir3062	Sectm1b	Ankib1	Grm7
Ttc24	Vmn1r69	Usp10	C8b
Srp9	Helz2	Usp10	Cd8b1
Cdx1	Olfr1051	Ifng	Gm25018
Olfr429	Tex55	Kcna2	Gm22291
Vmn1r85	1700031L13Rik	Paxbp1	Gm22109
Stmn4	Hand1	Fbp1	Gm23284
Crip3	Tmsb4x	Gm32921	Gm25212
Gm428	Adarb2	Mapre2	Gm26391
Olfr1009	Itga8	Apoa1	Klk1
Rprm	Calcr	Mir5709	8030451O07Rik
Qsox2	Olfr1205	Zfp169	Spem1
Gm11468	Wnt10b	Spaca1	Mir7059
Spaca5	Gm7788	Arhgap15	Ggt1
Tmem163	T	Olfr31	Col9a1
Ang5	Slco4c1	Defb12	1700074P13Rik
Ang3	Rbp4	Gm4737	Mpp2
Cct8	Hibch	Sult1c1	Erfe
Lyg1	Prr5l	A630076J17Rik	4933427D06Rik
Mcoln3	Olfr807	E030044B06Rik	Ffar1
Lrrtm3	Fam174a	1700022A22Rik	Pip5kl1

Ssu2	Wnt3a	Chd9	Wipf1
1700049L16Rik	Olfr8	Trim58	Olfr572
Snapc4	Vmn1r19	Rab26os	Ednra
Slc4a4	Gm2516	Traf7	Iqcf4
Vmn1r155	Ism1	4930529K09Rik	Casd1
Vmn1r107	Nedd1	4922502N22Rik	Hmga2-ps1
Vmn1r165	Cyp7b1	Zw10	1700031A10Rik
Vmn1r137	Mepe	Evl	Tent4b
Vmn1r130	Lrig3	Cnga1	Exoc3l2
Phf11b	Gpm6b	Camk1d	Arsg
Foxo4	Gm20491	Mmrn2	Lctl
Mkrn1	Olfr1040	Sncg	Mir7b
Gm10244	Polk	4930543N07Rik	Mboat4
Mkrn1	Col4a3bp	Olfr1426	Nelfb
Olfr732	Olfr1155	Gdf7	Stpg3
Aff2	Tut7	Olfr784	Mymk
Klk1b9	Iqcf5	Gm29687	Slc22a30
Olfr1284	Cd226	Evi5l	Pitx2
Gm21818	Lrp2bp	Dcaf7	Olfr996
Nmnat2	Tor1aip1	Obp2a	Leng9
Mir5098	Mre11a	Gpr63	Zfp558
Astn2	Il1b	Olfr1385	Inpp5j
Hrh4	Dmrtb1	Tex15	Cyp2c53-ps
4930529L06Rik	Atad2b	Sapcd1	Rnf169
Il1rl1	Hc	Fhad1os1	Prl7d1
1600014C23Rik	Ikzf1	Mrpl33	Olfr1255
F630040K05Rik	Nudc	Cpped1	Syt1
Ptk6	Trpa1	Olfr1511	Adcy7
Naaladl1	Ncf4	Vmn1r50	Hoxc12
Gm765	Josd2	Aebp2	Tmem106a
Glycam1	Aspdh	Ggt1	Lcp1
Col5a2	Ccdc124	Cox6b1	Olfr1426
Ifnl2	Metap2		

Genes with decreased PRDM16 binding to promoter after high glucose incubation

Fundc1	Tspyl2	Gm7102	Dnmt3a
Olfr851	Gm11596	Syt6	Trim43c
Olfr967	Gm29811	Slc2a10	Apbb1
Rfx6	Iltifb	Trim52	1700099I09Rik
B230303A05Rik	Olfr618	Vmn1r216	Ptpro
Ripor2	9330151L19Rik	Ptpru	Mfsd4a
Gm13498	Rbm48	Nme4	Rem2
Mcts1	Pex1	Srpx2	Tas2r116

Pitx2	Fam122b	C130036L24Rik	Tacr2
Olfr1186	Vmn1r200	2010009K17Rik	F8a
Mir12202	Atp1a3	4930413F20Rik	1500012K07Rik
Batf3	1700121L16Rik	Plekha7	Sv2b
Dctd	Clec10a	5730405O15Rik	Strbp
Xrcc1	Tbcc	Rbm47	Gm15412
Tmem189	Hrk	BC055402	Psg23
Entpd8	Cadps2	Krtap3-3	Apoa2
Mir6357	Rab5c	Zfp236	Slc36a4
4921518K17Rik	Lypd11	Mtpap	Ramp2
Tcaf3	1810044D09Rik	Ppp2r2c	Tnfrsf9
Esyt3	4930471M09Rik	Mir7658	Slc51a
4930529C04Rik	Gm10390	Mrgprb3	Lrrtm2
Dpy19l2	4930428D20Rik	Oc90	Ugt8a
Vmn1r28	Mroh9	Ankrd22	Lncenc1
Gm1965	Rlim	P2rx6	Sult2a1
Tbcel	Tex29	Rap1gap	Lap3
Nav1	Slamf8	BC016548	Fbxl22
Vax2os	Zbtb49	Lrrd1	Heyl
Adora3	Lyar	Lmo3	Tmem45a2
Suco	Fbln5	Cfap70	4930556J02Rik
Kdm3a	Ptchd3	Vps26b	1600002D24Rik
Sp6	Evc	Ncapd3	Olfr1380
Cilp	Crmp1	Mfsd7a	Olfr1444
Bfsp2	Smap1	Scgb1b24	Gm1966
Rnf125	Akap4	Tmprss5	Ankib1
Cdh22	Dynap	Tbrg1	Pramef17
Tbc1d30	Aqp9	Ptn	Shisa8
Itgam	Aven	1110058D11Rik	Mcm10
Disp3	Olfr720	Dnah7c	Vmn2r59
Extl1	4930448F12Rik	Mir6417	Zfp595
Klhl23	4930455F16Rik	Eif2c5	Nap1l5
D17Ertd648e	Gemin6	Trappc5	Tnnt2
1700030M09Rik	Ripk3	Adcy3	Apcdd1
Ppp3ca	Hsd3b1	Spdef	Gm10649
Gmnc	D330041H03Rik	Kcnn3	Gimap7
Casp14	Rnps1	Hspb7	Arg2
Gkap1	Elf1	Olfr214	Bach1
B3gnt3	Gm15694	Scn11a	Celsr2
Arl6	Dph1	Gm5741	Stox2
1700044C05Rik	Serpinb3b	1110017D15Rik	Cxcl3
Arhgef4	4930555B11Rik	Plscr3	Tfb1m
Oit3	BB557941	Hmgn2	1700102H20Rik

Galnt4	Dhx58	3000002C10Rik	Plekhb2
Tgm2	Mir12190	Nme6	Slc30a3
Hivep2	4930447J18Rik	Sgpp2	Ankrd35
Srpr	Ppp1r1b	Irgc1	1700065L07Rik
Foxred1	Setd6	Cep170b	4931417E11Rik
Olfr603	Gm10375	5033403H07Rik	AF357426
Eprs	Hba-a2	Arhgef16	Kmt2a
Olfr1275	Hba-a1	Spa17	Ech1
Kifc5b	Vit	Siae	Tmprss9
4930456L15Rik	Limk2	Atp13a3	Atg7
Map3k8	Pkd2l2	Rcan2	Slc3a1
4833419F23Rik	Ythdc1	Ube2v1	A630073D07Rik
Atp1a4	Ffar1	Syne1	1700011B04Rik
Zfp677	Itpr2	Kcnk18	Sim2
Gcfc2	Marveld2	Ntf5	Svip
Igf2	Mrpl35	BC006965	Strip1
Mesp2	A230072C01Rik	Cadps	Trim33
Rev3l	Uxt	Tmem201	Mir6982
E130307A14Rik			

Supplementary Table 3: The TRPA1 promoter sequences used in the luciferase reporter assays

P4-1
CACTCCTTGGGGACAGCAGTATGCATTTTATTCCATTGAGCCTTTTGGAA
TTGGAAATGTGCCTTCATTAGATCACCACCTTTACTTTTTTCTTGTATAA
TACAAAGTGCGAATGTATCAGATAGCCCAGCTCTGAGCTTCATAAAGTGA
TTCTCATGCTCATTCCCTCACCAGCTCCAGAGTATCTCTACCCTATGAAC
ATGACTTTGACTATGCATGTCCTTATGTTATGTAGGTAGTGTTGTTTTGA
GTATTTAATGTCTTTTTTTCTATTAGCTATTTTTTCCCAATGGATGTTAT
GCTTAATTTACAAGATAAATTGCACTGTGTGCCCATCTAGTGTAGGGCGT
CTGACAGTCCATCTTATTTTGTAGACTCTTATATTTAACTGGTCTAAGAG
ATGGGTATCTTAACGGTTTCCACCTTCTTGCCGCCACATACATTCAGCGC
AAGTTCAACACCCAGTGTTGCATAGCAACTTGTCATATTATAAACCCAGA

P4-2
CACTCCTTGGGGACAGCAGTATGCATTTTATTCCATTGAGCCTTTTGGAA
TTGGAAATGTGCCTTCATTAGATCACCACCTTTACTTTTTTCTTGTATAA
TACAAAGTGCGAATGTATCAGATAGCCCAGCTCTGAGCTTCATAAAGTGA
TTCTCATGCTCATTCCCTCACCAGCTCCAGAGTATCTCTACCCTATGAAC
ATGACTTTGACTATGCATGTCCTTATGTTATGTAGGTAGTGTTGTTTTGA
GTATTTAATGTCTTTTTTTCTATTAGCTATTTTTTCCCAATGGATGTTAT

GCTTAATTTACAAGATAAATTGCACTGTGTGCCCATCTAGTGTAGGGCGT
CTGACAGTCCATCTTATTTTGTAGACTCTTATATTTAACTGGTCTAAGAG

P4-3
CACTCCTTGGGGACAGCAGTATGCATTTTATTCCATTGAGCCTTTTGGAA
TTGGAAATGTGCCTTCATTAGATCACCACCTTTACTTTTTTCTTGTATAA
TACAAAGTGCGAATGTATCAGATAGCCCAGCTCTGAGCTTCATAAAGTGA
TTCTCATGCTCATTCCCTCACCAGCTCCAGAGTATCTCTACCCTATGAAC
ATGACTTTGACTATGCATGTCCTTATGTTATGTAGGTAGTGTTGTTTTGA
GTATTTAATGTCTTTTTTTCTATTAGCTATTTTTTCCCAATGGATGTTAT

P4-4
CACTCCTTGGGGACAGCAGTATGCATTTTATTCCATTGAGCCTTTTGGAA
TTGGAAATGTGCCTTCATTAGATCACCACCTTTACTTTTTTCTTGTATAA
TACAAAGTGCGAATGTATCAGATAGCCCAGCTCTGAGCTTCATAAAGTGA
TTCTCATGCTCATTCCCTCACCAGCTCCAGAGTATCTCTACCCTATGAAC

P4-5
CACTCCTTGGGGACAGCAGTATGCATTTTATTCCATTGAGCCTTTTGGAA
TTGGAAATGTGCCTTCATTAGATCACCACCTTTACTTTTTTCTTGTATAA

Supplementary Table 4. The basic clinical information of DKD (early stage) and DKD (late stage) patients

Variable	DKD (early stage) (n=3)	DKD (late stage) (n=3)
**Age(years) (mean)[SD]**	**51.3±1.70**	**56±6.48**
**Gender (Male:Female)**	**1 ： 2**	**3 ： 0**
**Serum Glucose(mmol/L) (mean)[SD]**	**9.43±0.17**	**10.76±0.88**
**eGFR ml/min/1.73 m^2(mean) [SD]**	**73.07±7.23**	**20.80±1.67#**
**Urine Protein**	**+:2**	**+++：3**
	**++:1**	

Note: Data are expressed as mean ± SD (n = 3). #p < 0.05, DKD (early stage) groups versus DKD (late stage) groups.
